# Key determinants of target DNA recognition by retroviral intasomes

**DOI:** 10.1186/s12977-015-0167-3

**Published:** 2015-04-30

**Authors:** Erik Serrao, Allison Ballandras-Colas, Peter Cherepanov, Goedele N Maertens, Alan N Engelman

**Affiliations:** Department of Cancer Immunology and AIDS, Dana-Farber Cancer Institute, Boston, MA USA; Division of Infectious Diseases, Imperial College London, London, UK; Clare Hall Laboratories, The Francis Crick Institute, London, UK

**Keywords:** Retrovirus, Integrase, DNA flexibility, Dinucleotide steps, Integration sites, Nucleosomes, BET proteins

## Abstract

**Background:**

Retroviral integration favors weakly conserved palindrome sequences at the sites of viral DNA joining and generates a short (4–6 bp) duplication of host DNA flanking the provirus. We previously determined two key parameters that underlie the target DNA preference for prototype foamy virus (PFV) and human immunodeficiency virus type 1 (HIV-1) integration: flexible pyrimidine (Y)/purine (R) dinucleotide steps at the centers of the integration sites, and base contacts with specific integrase residues, such as Ala188 in PFV integrase and Ser119 in HIV-1 integrase. Here we examined the dinucleotide preference profiles of a range of retroviruses and correlated these findings with respect to length of target site duplication (TSD).

**Results:**

Integration datasets covering six viral genera and the three lengths of TSD were accessed from the literature or generated in this work. All viruses exhibited significant enrichments of flexible YR and/or selection against rigid RY dinucleotide steps at the centers of integration sites, and the magnitude of this enrichment inversely correlated with TSD length. The DNA sequence environments of *in vivo-*generated HIV-1 and PFV sites were consistent with integration into nucleosomes, however, the local sequence preferences were largely independent of target DNA chromatinization. Integration sites derived from cells infected with the gammaretrovirus reticuloendotheliosis virus strain A (Rev-A), which yields a 5 bp TSD, revealed the targeting of global chromatin features most similar to those of Moloney murine leukemia virus, which yields a 4 bp duplication. *In vitro* assays revealed that Rev-A integrase interacts with and is catalytically stimulated by cellular bromodomain containing 4 protein.

**Conclusions:**

Retroviral integrases have likely evolved to bend target DNA to fit scissile phosphodiester bonds into two active sites for integration, and viruses that cut target DNA with a 6 bp stagger may not need to bend DNA as sharply as viruses that cleave with 4 bp or 5 bp staggers. For PFV and HIV-1, the selection of signature bases and central flexibility at sites of integration is largely independent of chromatin structure. Furthermore, global Rev-A integration is likely directed to chromatin features by bromodomain and extraterminal domain proteins.

**Electronic supplementary material:**

The online version of this article (doi:10.1186/s12977-015-0167-3) contains supplementary material, which is available to authorized users.

## Background

The integration of a DNA copy of the viral RNA genome into a host cell chromosome is a critical step in the retroviral lifecycle. Retroviruses accordingly encode for an integrase (IN) enzyme, which is a specialized DNA recombinase. Integration begins with the formation of the intasome nucleoprotein complex, which consists of an IN tetramer assembled on the ends of the linear viral DNA (vDNA) [1-3]. The two inner subunits of the tetramer cleave the vDNA ends adjacent to invariant 5′-CA-3′ dinucleotides to yield reactive CA_OH_-3′ hydroxyl groups [3-8]. The intasome is transported from the cytoplasm to the nucleus as part of a large assembly of viral and host proteins known as the preintegration complex [9-11]. In the nucleus the intasome engages host cell chromatin to form the target capture complex (TCC) [3,12]. The inner subunits of the IN tetramer utilize the vDNA CA_OH_-3′ termini to cleave both strands of the target DNA (tDNA) in a staggered fashion, at the same time joining the vDNA ends to tDNA 5′-phosphates [13]. The resulting DNA recombination intermediate contains free vDNA 5′ ends abutting single stranded gaps in the tDNA, which vary in length from four to six nucleotides, depending on the retrovirus [14-17]. The single-stranded gaps are repaired by host cell enzymes to yield a target site duplication (TSD) of 4–6 bp flanking the provirus.

Several features of the animal cell genome, from the tDNA sequence at the site of integration to higher-order chromatin structure, can influence the selection of retroviral integration sites (see [18] for a recent review). Seven different genera, alpha through epsilon, lenti, and spuma, comprise the Retroviridae family, and preferential targeting of structural chromatin features is most evident for the lenti- and gammaretroviruses. Lentiviruses preferentially integrate along the bodies of actively transcribed genes [19], whereas the gammaretroviruses favor transcriptional start sites (TSSs) and active enhancer regions [20-22]. These preferences are in large part governed by interactions between IN proteins and cognate cellular factors [18]. The lentiviral IN-binding protein lens epithelium-derived growth factor (LEDGF)/p75 directs integration to active genes [23-26], whereas bromodomain and extraterminal domain (BET) proteins BRD2, 3, and 4 interact with Moloney murine leukemia virus (MoMLV) IN to affect TSS-proximal integration [27-29]. Viruses from the other profiled genera – integration site preferences of epsilonretroviruses have not been reported – show less propensity to target chromatin-specific features than do either the lentiviruses or gammaretroviruses, with the betaretrovirus mouse mammary tumor virus (MMTV) displaying the least selectivity of all [30].

Analyses of retroviral integration sites revealed weak palindromic tDNA sequence consensuses at the sites of vDNA joining [14-17,31]. A palindromic consensus implies dyadic symmetry within the IN nucleoprotein complex that engages tDNA, and crystallographic analysis of the prototype foamy virus (PFV) TCC revealed key features of the inner IN dimer within the tetramer that dictate the selection of the consensus PFV integration site (−3)KWK\*VYRB*MWM(+6) (written using International Union of Biochemistry base codes; the backslash indicates the position of vDNA plus-strand joining, and the italics mark the TSD, which is 4 bp for PFV) [12]. The tDNA is accommodated in a severely bent conformation, with the major groove widened such that the dinucleotide at the center of the integration site (YR) is unstacked. Given the relatively weak nature of nucleotide specificity at integration sites, it was not surprising that a number of IN main chain amide groups interacted with the tDNA backbone in the TCC structure. In addition, base specific contacts were observed for PFV IN residues Ala188, which resides in the catalytic core domain (CCD), and Arg329, which is part of the IN C-terminal domain. Ala188 in particular interacted with bases that lay 3 positions upstream from the points of vDNA joining, whereas Arg329 interacted with bases at either edge of the integration site, as well as those at symmetric nucleotide positions −2 and +5 [12].

The variety of dinucleotide steps differ in their propensity to support distortion of a DNA double helix, which reflects their inherent base stacking interactions [32]. Pyrimidine-purine (YR) and RY steps are the most and least distortable, respectively, whereas YY and RR display an intermediary level of flexibility. The strongly preferred YR at the center of PFV integration sites accommodates the sharp tDNA bend required for integration. The consensus target site sequence (−3)TDG\*(G/V)TWA(C/B)*CHA(+7) for human immunodeficiency virus type 1 (HIV-1) was subsequently shown to harbor the dinucleotide signature motif (0)RYXRY(+4), which selects against rigid RY dinucleotides at the center of integration sites (due to the odd number of bp between the sites of HIV-1 DNA joining, the location of the integration site center encompasses two overlapping positions: nucleotides +1 and +2 and nucleotides +2 and +3) [33].

Inherent curvature or bendability of DNA substrates positively correlates with frequency of integration targeting [34-36], and tDNA deformed by the binding of nucleosomes [37-39] or other DNA bending proteins [34,40] can be utilized preferentially by IN over naked DNA *in vitro*. Nucleosomes are favored sites for integration during MoMLV and HIV-1 infection [41-45]. Moreover, A/T-rich sequences that emanate outward from the central, local palindrome at the sites of vDNA insertion exhibit periodicity coincident with the outward-facing major grooves on the nucleosome surface [34,37,38,42-44]. However, because PFV [46] and HIV-1 [33] integration *in vivo* and using naked plasmid tDNA substrates *in vitro* generated similar palindrome signatures, the forces that govern the selection of particular bases at the sites of integration appear for the most part independent of tDNA chromatinization.

In this study we extended dinucleotide step analysis of retroviral integration sites to a total of 12 viruses. We find that central flexibility is a conserved feature and that it inversely correlates with the length of TSD. By comparing integration sites in naked plasmid or cellular tDNA to those generated during PFV and HIV-1 infection, we confirm that central flexibility and local nucleotide preferences are for the most part independent of nucleosome content. Furthermore, we report the integration site preferences of reticuloendotheliosis virus strain A (Rev-A) in infected cells, which paralleled those of previously reported gammaretroviruses despite the fact that Rev-A integration generates a 5 bp duplication of tDNA. Thus, Rev-A integration distribution mirrored that of MoMLV, and we accordingly show that Rev-A IN interacts with and is catalytically stimulated by a portion of the MoMLV integration host cofactor BRD4 that contains the IN-interacting extraterminal (ET) domain. Akin to MoMLV [27-29], IN binding to BET proteins likely directs Rev-A integration to chromatin features such as TSSs.

## Results

### Analytic strategy

In light of the similarity in the selection for central flexibility at sites of PFV and HIV-1 integration, we extended dinucleotide frequency analyses to a total of 12 retroviruses. Considering that retroviral TSDs vary from 4 to 6 bp, we analyzed four viruses that generate 4 bp duplications, four that generate 5 bp duplications, and four that generate 6 bp duplications. Viruses from two to three different genera comprise each of these subsets (Table 1). Four bp TSDs are yielded by the spumavirus PFV [47,48] as well as the gammaretroviruses MoMLV [49,50], porcine endogenous retrovirus (PERV) [51,52], and xenotropic murine leukemia virus-related virus (XMRV) [53,54]. The alpharetrovirus avian sarcoma-leukosis virus (ASLV) [55,56], deltaretrovirus human T-lymphotropic virus type 1 (HTLV-1) [57,58], and betaretroviruses human endogenous retrovirus K family (HERV-K) [59] and MMTV [60] yield 6 bp TSDs. In addition to HIV-1 [61,62], the lentiviruses simian immunodeficiency virus (SIV) [63] and equine infectious anemia virus (EIAV) [64], as well as the gammaretrovirus Rev-A [65,66], yield 5 bp TSDs.

**Table 1:** **Retroviruses included in this study**

**Virus (genus)**	**TSD (bp)**	**Reference(s)**	**Number of sequences** ^***a***^
PFV (spuma)	4	[92,93]	2,924
MoMLV (gamma)	4	[44]	53,463
PERV (gamma)	4	[94]	1,668
XMRV (gamma)	4	[44,53]	5,487
EIAV (lenti)	5	[24,64]	1,172
HIV-1 (lenti)	5	[87]	335,968
Rev-A (gamma)	5	This study	834
SIV (lenti)	5	[95,96]	168
ASLV (alpha)	6	[97-99]	916
HERV-K (beta)	6	[100]	1,071
HTLV-1 (delta)	6	[17,101,102]	6,820
MMTV (beta)	6	[103]	178,574

^*a*^All sequences derived from virus-infected cells.

Perusal of the literature revealed that the number of reported integration site datasets from virus-infected cells ranged from a small handful for Rev-A [65] to several million for MoMLV [21,22]. Rev-A is of particular interest, as it is a gammaretrovirus with a 5 bp TSD; all other gammaretroviruses yield 4 bp TSDs [14,15,17]. We accordingly initiated this study by determining the sequences of 834 unique integration sites from HEK293T cells infected with a Rev-A viral vector by ligation-mediated PCR. The targeting preferences of Rev-A for genomic annotations such as genes, TSSs, CpG islands, and gene density are described toward the end of the Results section. As the HEK293T DNA was fragmented by digestion with restriction endonucleases AvrII, NheI, and SpeI, a matched random control (MRC) of 282,824 unique sites was produced by selecting random positions in proximity of these restriction sites in human genome build 19 (hg19). Sequences extracted from GenBank (http://www.ncbi.nlm.nih.gov/genbank/) or obtained from the authors of prior studies yielded datasets for the remaining 11 viruses that encompassed from ~170 to 336,000 unique integration sites (Table 1).

**Figure 1:**
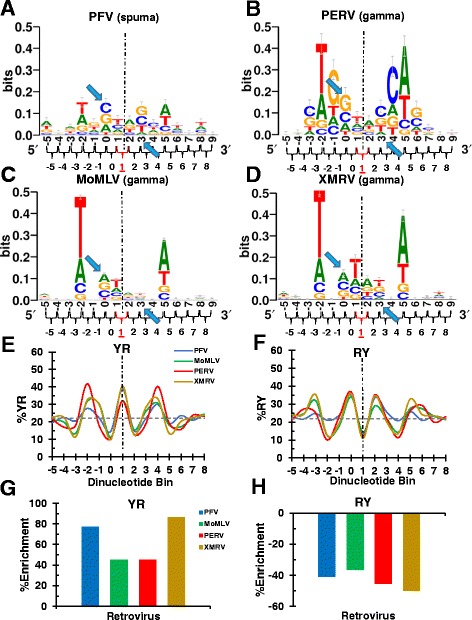
Sequence logos and dinucleotide step analysis of integration sites with 4 bp TSDs. **(A-D)** The height of each individual base at a given position is proportional to the frequency of the corresponding nucleotide within the sequences represented by the logos, and the height of each stack of base logos reflects the level of conservation at that position. **(E)** Percent YR utilization across the integration sites from panels **A-D** is shown relative to the calculated random value of 22% (dotted gray horizontal line). **(F)** Same as in panel **E**, except that the graph depicts RY utilization across the integration sites. Statistical analysis of panel **E** and **F** results are shown in Additional file 2: Figure S2 panels **A** and **B**, respectively. **(G and H)** The percent of YR (panel G) and RY (panel H) enrichment for each virus compared to random.

**Figure 2:**
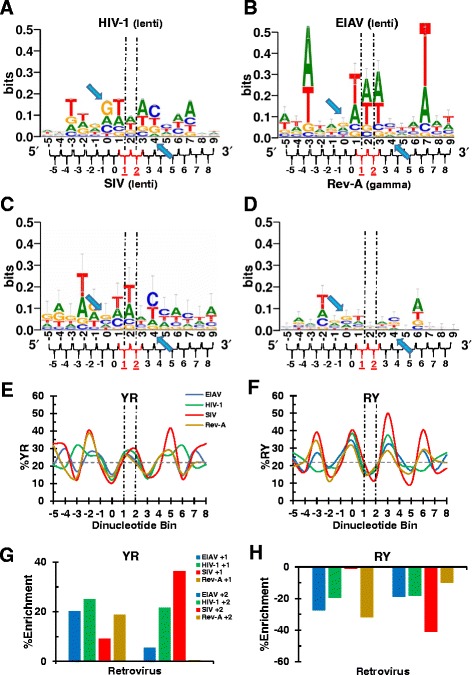
Sequence logos and dinucleotide step analysis of integration sites from retroviruses that yield 5 bp TSDs. Sequence logos for HIV-1 **(A)**, EIAV **(B)**, SIV **(C)**, and Rev-A **(D)**. **(E)** YR step analysis for the integration sites depicted in panels **A-D**. **(F)** Same as in panel **E**, except RY dinucleotide frequencies were calculated. Statistical analyses of panel **E** and **F** results are depicted in Additional file 2: Figure S2 panels **C** and **D**, respectively. Percent YR and RY enrichment for each virus compared to random is shown in **(G)** and **(H)**, respectively. Other labeling is as in Figure 1.

**Figure 3:**
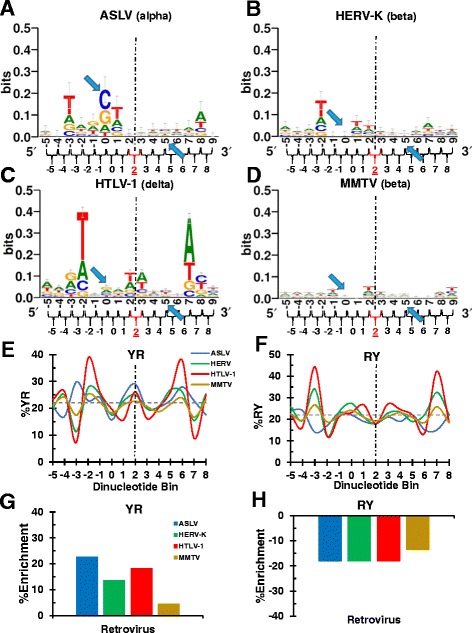
Sequence logos and YR/RY dinucleotide selectivities of viral integration sites with 6 bp TSDs. Sequence logos are shown for conglomerate integration sites of ASLV **(A)**, HERV-K **(B)**, HTLV-1 **(C)**, and MMTV **(D)**. **(E)** YR frequency utilization across the integration sites of viruses depicted in panels **A-D**. **(F)** Same as in panel **E**, except the plot is for RY dinucleotide utilization. Statistical analyses of panel **E** and **F** results are shown in Additional file 2: Figure S2 panels **E** and **F**, respectively. The percent YR and RY enrichment for each virus compared to random is in **(G)** and **(H)**, respectively. Other labeling is as in Figure 1.

Sequence logos [67] were compiled to provide a visualization of base preferences at each nucleotide position at and surrounding the points of vDNA joining for the 12 different viruses (see Figures 1, 2 and 3, A-D). The position of vDNA insertion on the tDNA plus strand by convention was designated as 0, with upstream and downstream nucleotide positions extending in the negative and positive directions, respectively. As the most significant retroviral tDNA base preferences exist within and closely adjacent to the TSD [42] (the boundaries of which are marked by blue arrows in the sequence logos), we initially limited our analysis to base positions −5 through +9, or a total of 15 nucleotides. The statistical significance of nucleotide frequencies for each virus at each tDNA base position, which was compared to the MRC that was generated for the Rev-A integration site analysis, is presented in Additional file 1: Figure S1. These frequencies were calculated as observed-to-expected ratios and were thus normalized for human genomic DNA G/C content. The sequence logos by contrast should be primarily considered as visual aids because they depict raw base frequencies without normalization. The vast majority of positions within the 15 base windows displayed statistically significant variance from random across the viral integration sites (Additional file 1: Figure S1). For the dinucleotide step analysis, successive nucleotide positions were binned into groups of two; dinucleotide bin numbers are annotated below the sequence logo x-axes in Figures 1, 2 and 3, panels A-D. The frequencies of YR and RY dinucleotide usage at each bin position were compared to random frequencies using Fisher’s exact test (Additional file 2: Figure S2).

### Dinucleotide step analysis of viral integration sites with 4 bp TSDs

Symmetric base preferences project outward from the center of retroviral integration sites [14-17]. By convention, the center of an integration site with a 4 bp TSD is designated between nucleotide positions +1 and +2, which coincides with dinucleotide bin position +1 (Figure 1, vertical dotted line). As expected, PFV integration selected for flexible YR dinucleotides at this position [12] (Figure 1E and Additional file 2: Figure S2A). Perusal of the sequence logos indicated unique nucleotide signatures across the integration sites of viruses that generate 4 bp TSDs (Figure 1A-D). Nevertheless, commonalities among nucleotide and dinucleotide content were evident across these sites. Within the TSD, thymidine and adenosine were disfavored at symmetric positions 0 and +3, respectively. By contrast, outside of the TSD window T and A were preferred at symmetric positions −2 and +5, respectively (Additional file 1: Figure S1). Moreover, there was strong consensus for YR selectivity at the central dinucleotide step (Figure 1E and Additional file 2: Figure S2A). The calculated random frequency of YR dinucleotide occurrence in the human genome is 22% (Figure 1E, grey dashed horizontal line). YR utilization at bin position +1 by PFV, MoMLV, PERV, and XMRV were on average increased by 63% relative to this value (Figure 1G), equating to highly significant differences (*P* values ranging from 5 x 10^−21^ for PERV to >2.2 x 10^−308^ for MoMLV; Additional file 2: Figure S2A). On the contrary, all four viruses displayed strong selection against central RY steps, with an average value depressed by 43% relative to the expected value (Figure 1F, H, and Additional file 2: Figure S2B; *P* values of 1.9 x 10^−27^ for PERV to >2.2 x 10^−308^ for MoMLV).

### Dinucleotide step analysis of viruses that yield 5 bp TSDs

Because viruses like HIV-1 join their vDNA ends across an odd number of tDNA bp, the center of their integration sites falls squarely on nucleotide position +2, which is a common element of dinucleotide bins +1 and +2 (Figure 2A-D). As recently elucidated the consensus sequence (0)RYXRY(+4), which resides at the center of HIV-1 integration sites [33], ensures for either YR or YY at nucleotide positions +1 and +2, and for YR or RR at nucleotide positions +2 and +3. Therefore, HIV-1 on average selects for a flexible YR step at one of the two central dinucleotide positions while strongly selecting against rigid RY steps. The tDNA sequences surrounding the integration sites of viruses that yield 5 bp TSDs were generally dissimilar from one another (Figure 2A-D). However, as was the case for the viruses that yield 4 bp TSDs, commonalities were evident among the sites that harbor 5 bp TSDs. HIV-1, EIAV, SIV, and Rev-A significantly disfavored T/A bases at the positions that delineate the external boundaries of the TSD, which in this case is positions 0 and +4, respectively (Additional file 1: Figure S1). All four viruses also displayed the preference for (0)RYXRY(+4) at the duplicated region and the symmetric preference for T/A adjacent to the TSD that was exhibited by 4-bp duplicating viruses. The positioning of this preference relative to the TSD window however varied among this set of viruses, falling two bases exterior to the TSD for SIV and Rev-A but three bases exterior for HIV-1 and EIAV (Additional file 1: Figure S1; Figure 2A-D).

Perhaps reflecting the fact that the center of these integration sites is spread over two dinucleotides, variable preference for YR/RY selectivity was apparent at the two central positions. At dinucleotide bin position +1, EIAV, HIV-1, and Rev-A each exhibited a similar enrichment for YR utilization, with an average relative increase of ~20% from the random value (Figure 2E, G, and Additional file 2: Figure S2C; *P* values from 5 x 10^−3^ for Rev-A to >2.2 x 10^−308^ for HIV-1). Although SIV also trended toward YR enrichment at bin position +1, this increase was not statistically different from random (Additional file 2: Figure S2C, *P* = 0.46). As reported [33], the enrichment for YR utilization at bin position +2 by HIV-1 was statistically significant (Figure 2E, G, and Additional file 2: Figure S2C; *P* >2.2 x 10^−308^). While EIAV and SIV also trended toward YR enrichment at bin position +2, the difference only achieved statistical significance for SIV (*P* = 0.02). Rev-A by contrast did not exhibit YR enrichment at dinucleotide bin position +2. In terms of RY selectivity (Figure 2F, H, and Additional file 2: Figure S2D), EIAV and HIV-1 similarly avoided the rigid step at bin positions +1 and +2, averaging ~20% decreases from random (Additional file 2: Figure S2D, *P* values ranging from 5 x 10^−4^ for EIAV at bin position +2 to >2.2 x 10^−308^ for HIV-1 at both positions). While trending toward selection against RY at both dinucleotide positions, Rev-A registered as statistically different from random only at position +1 (*P* = 10^−6^) while SIV registered as different only at position +2 (*P* = 0.002).

### Dinucleotide step analysis of viral integration sites with 6 bp TSDs

The center of the integration site for viruses that yield 6 bp TSDs lies between nucleotide positions +2 and +3, which coincides with dinucleotide bin position +2 (Figure 3A-D). These integration sites on average yielded sequence logos with lower information content scores than the viruses that create 4 bp and 5 bp TSDs (compare Figure 3 to Figures 1 and 2). As with the previously discussed integration sites, T/A tended to be disfavored at the inner edges of the TSD window, though this was more evident for the sites generated by ASLV, HERV-K, and HTLV-1 than it was for MMTV (Additional file 1: Figure S1). ASLV, HERV-K, and HTLV-1 also revealed preference for T/A outside of the TSD window, two bases removed from the window for HERV-K and HTLV-1 but three bases removed for ASLV (Additional file 1: Figure S1),

All four viruses exhibited a significant enrichment for YR utilization at the center of their integration sites (Figure 3E, G, and Additional file 2: Figure S2E; *P* values ranged from 0.02 for HERV-K to 1.1 x 10^−15^ for HTLV-1). The selection against RY utilization at dinucleotide bin position +2 by these viruses was also significant (Figure 3F, H), yielding *P* values that ranged from 3.2 x 10^−4^ for HERV-K to 8.9 x 10^−110^ for MMTV (Additional file 2: Figure S2F).

### Target DNA base preferences and central flexibility are determined by IN independent of nucleosome content

Integration sites on nucleosomal tDNA map to positions of DNA major groove distortion *in vitro* [35,37-39] and during virus infection [41-44]. Prior work with PFV [46] and HIV-1 [33] revealed that similar bases were selected in cells and when using the respective purified IN protein with naked tDNA *in vitro*, implying that nucleosome structure may not grossly influence the selection of tDNA bases at the sites of vDNA joining. However, the naked tDNA used in these studies was supercoiled plasmid with relatively low sequence diversity and little-to-no capacity to position native nucleosomes. Therefore, we accessed a panel of 22,117 unique integration sites from a reaction that utilized recombinant PFV intasomes and deproteinized human DNA [39], which served as an optimally diverse, nucleosome-free tDNA substrate.

**Figure 4:**
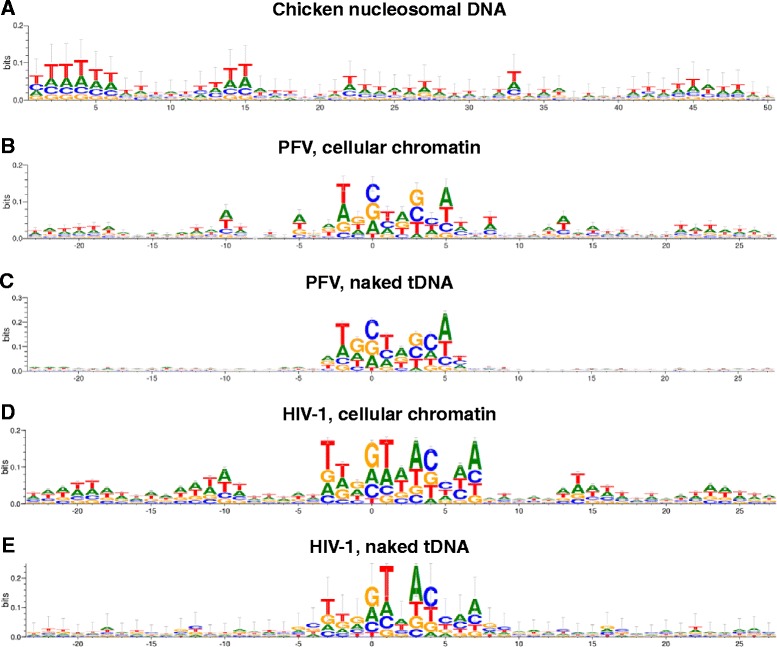
Sequence logos for PFV and HIV-1 integration sites in nucleosome-free versus chromatinized tDNA. **(A)** The logo illustrates the average nucleotide sequence of the first 50 nucleotides of center-aligned nucleosomal DNA sequences isolated from chicken erythrocytes [68]. **(B)** PFV integration sites derived from virus-infected cells [92,93]. **(C)** Integration sites from recombinant PFV intasomes and deproteinized cellular DNA. **(D)** HIV-1 integration sites from virus-infected cells [87]. **(E)** Concerted HIV-1 integration sites from recombinant HIV-1 IN and naked pGEM9Zf(−) plasmid DNA [33].

**Figure 5:**
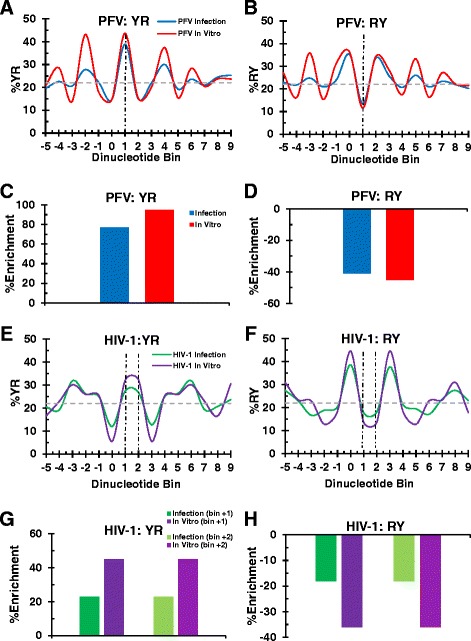
Flexibility profiles for PFV and HIV-1 integration sites in nucleosome-free versus chromatinized tDNA. **(A and B)** YR and RY frequency charts, respectively, for PFV integration sites into deproteinized genomic DNA (PFV *in vitro*) and from virus infection. Vertical dotted black line represents central dinucleotide step(s), and horizontal dotted grey line represents the MRC frequency of YR/RY utilization. **(C and D)**) Bar graphs illustrating the percent YR and RY enrichment, respectively, at the central dinucleotide step relative to MRC values. **(E and F)** YR and RY frequency charts, respectively, for HIV-1 integration sites into naked plasmid DNA (HIV-1 *in vitro*) and from virus infection. **(G and H)** Bar graphs illustrating the percent YR and RY enrichment, respectively, at the central dinucleotide steps compared to MRC.

**Figure 6:**
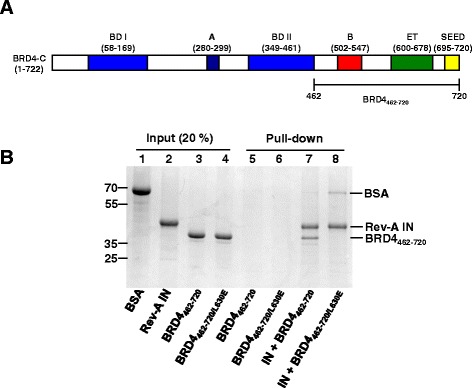
BRD4 protein and interaction with Rev-A IN. **(A)** Schematic of human BRD4 isoform C (NCBI reference sequence NP_055114.1) highlighting various protein domains and the 426–720 fragment used in this study. **(B)** Ni-NTA pull-down of purified BRD4_462–720_ by His_6_-tagged Rev-A IN. Migration positions of standards (in kDa) are labeled to the left. **Lanes 1–4**: 20% reaction input of the indicated proteins. **Lanes 5 and 6**: the indicated BRD4_462–720_ protein was incubated with Ni-NTA beads in the absence of Rev-A IN. **Lanes 7 and 8**: the indicated BRD4_462–720_ protein was incubated with Rev-A IN-bound beads. The gel is representative of results obtained from three independent experiments.

**Figure 7:**
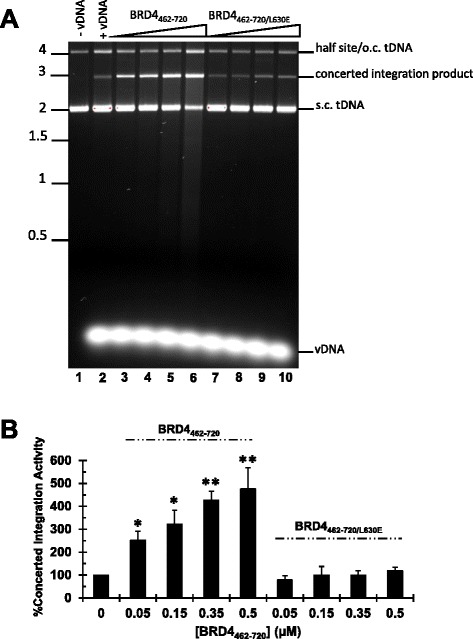
Concerted integration activity of Rev-A IN. **(A)** Ethidium bromide stained image of Rev-A IN integration reactions in the presence of increasing concentrations of indicated BRD4_462–720_ protein. Migration positions of standards (in kb) are shown to the left, and the positions of single vDNA end, or half-site, and concerted vDNA integration products, as well as supercoiled (s.c.) and open circular (o.c) forms of the plasmid tDNA, are to the right. **Lanes 1 and 2**: Rev-A IN and tDNA incubated without vDNA or with vDNA, respectively. **Lanes 3–6:** increasing concentrations (0.05, 0.15, 0.35, 0.5 μM) of BRD4_462–720_ incubated with Rev-A IN plus vDNA and tDNA. **Lanes 7–10**: same as lanes 3–6 but with BRD4_462–720/L630E_. **(B)** Strand transfer activities for three independent experiments ± standard error of the mean, measured by quantification of DNA band intensity. The results were normalized to the level of Rev-A IN concerted integration activity in the absence of BRD4_462–720_ proteins, which was set to 100%. *P* values of <0.05 and <0.01 are indicated by * and **, respectively, as determined by one-tailed t-test.

The window of sequence logo analysis was extended from 15 bp (Figures 1, 2 and 3) to 50 bp (Figure 4) to assess signature tDNA sequences preferentially bound by nucleosomes, which show on average a 10.6 bp periodicity for A/T-rich sequences [68] (Figure 4A). Comparing integration sites derived from PFV infected cells (Figure 4B) to those generated *in vitro* with deproteinized cellular DNA (Figure 4C) confirmed that the preference for local tDNA sequences at the sites of virus insertion were in large part generated independent of nucleosome content. Re-analyzing 122 previously-reported *in vitro* concerted integration events [33] to sites derived from virus infected cells (Table 1) recapitulated the finding that the preference for local tDNA sequence at the sites of HIV-1 integration was independent of nucleosome content (Figure 4D, E). Both PFV and HIV-1 cell-based datasets exhibited cyclical A/T-rich sequences that extended symmetrically outward from the TSD with approximate 10 bp periodicity (Figure 4B, D), as described previously for HIV-1 [42]. These cyclical base preferences, which were absent from *in vitro* datasets (Figure 4C, E), and reminiscent of the A/T-rich periodicity exhibited by nucleosome-bound DNA (Figure 4A), indicated that PFV and HIV-1 IN select for their preferred local tDNA sequences in the context of nucleosomal DNA during virus infection [41,42] (Figure 4).

PFV and HIV-1 selected for marginally distinguishable flexibility profiles at integration sites in naked tDNA *in vitro* versus cellular DNA (Figure 5). As discussed above, raw frequencies of YR enrichment and RY avoidance for PFV at dinucleotide +1 equated to 39% and 13%, respectively (Figure 5A, B, blue curves). These values corresponded to a 77% increase in YR utilization and a 41% decrease in RY utilization relative to the MRC values (Figure 5C, D). The bias for YR utilization and against RY utilization at the center of integration sites was marginally greater when using recombinant PFV IN and naked cellular DNA than they were for virus-infected cells. Specifically, IN selected for YR and RY frequencies of 43% and 12% (Figure 5A, B), equating to a 95% increase and a 45% decrease from random, respectively (Figure 5C, D). These same trends also applied to HIV-1. Raw YR frequencies at central bins +1 and +2 were 27%/27% for virus and 32%/32% for recombinant IN protein (Figure 5E), and RY frequencies were 18%/18% for virus and 14%/14% for recombinant IN (Figure 5F). Comparing these raw frequencies to the MRC, YR was enriched by 23%/23% for virus and 45%/45% for recombinant IN, while RY was avoided by 18%/18% for virus and 36%/36% for recombinant IN (Figure 5G, H).

### Genomic distribution of retroviral integration sites

Using various parameters linked to integration that include IN amino acid sequence, targeting of cellular chromatin features, and length of TSD, prior studies have phylogenetically linked subgroups of retroviral genera together [17,64]. We recently questioned the general applicability of this approach, as MoMLV and Rev-A, which are both gammaretroviruses, display similar tDNA base preferences but yield 4 and 5 bp TSDs, respectively [66]. It was therefore of interest to test if Rev-A integration distribution in cellular chromatin resembled that of MoMLV and/or other retroviruses. We accordingly mapped all of the integration sites used in this study, which included 834 unique sites from Rev-A-infected cells, with respect to several genomic annotations including RefSeq genes, CpG islands, TSSs, and gene density (Table 2). The statistical relevance of observed frequencies versus the MRC were determined by Fisher’s exact test for RefSeq genes, CpG islands, and TSSs and by Wilcoxon rank-sum test for gene density (*P* values listed in Additional file 3: Table S1).

**Table 2:** **Genomic distribution of retroviral integration sites**

**Library**	**Unique sites**	**Within Refseq genes (%)**	**Within 5 kb (+/− 2.5 kb) of CpG (%)**	**Within 5 kb (+/− 2.5 kb) of TSS (%)**	**Average gene density per Mb (+/− 0.5 Mb) of integration sites**
PFV	2,924	1,180 (40.4)	450 (15.4)	425 (14.5)	10.8
PFV (*in vitro*)	22,117	10,506 (47.5)	1,349 (6.1)	1,150 (5.2)	10.3
MoMLV	53,463	30,865 (57.7)	15,157 (28.4)	14,870 (27.8)	15.4
PERV	1,668	936 (56.1)	811 (48.6)	676 (40.5)	23.4
XMRV	5,487	3,086 (56.2)	1,022 (18.6)	1,100 (20.1)	14.5
EIAV	1,172	689 (58.8)	70 (6.0)	69 (5.9)	12.1
HIV-1	335,968	250,552 (74.6)	18,871 (5.6)	13,882 (4.1)	19.9
Rev-A	834	460 (55.2)	216 (25.9)	216 (25.9)	15.7
SIV	168	142 (84.5)	4 (2.4)	3 (1.8)	17.8
ASLV	916	320 (54.7)	55 (9.4)	44 (7.5)	11.3
HERV-K	1,071	541 (50.5)	131 (12.2)	108 (10.1)	17.8
HTLV-1	6,820	3148 (49.8)	523 (8.3)	467 (7.4)	10.7
MMTV	178,574	72,035 (40.3)	7,131 (4.0)	6,957 (3.9)	8.3
MRC	282,824	129,287 (45.7)	12,914 (4.6)	13,942 (4.9)	9.2

The results of our analyses of 11 previously profiled retroviruses are in line with those of the prior reports (see Table 1 for the list of references). Distributions relative to CpG islands and TSSs were calculated by counting sites that fell within a 5 kb window (+/− 2.5 kb) of these features, while average gene density was calculated by counting the number of RefSeq genes falling within a 1 Mb window (+/− 500 kb) of each integration site, and then averaging this value for the entire dataset. Our MRC dataset revealed that 45.7% of human DNA comprised RefSeq genes (Table 2). Most of the viruses targeted RefSeq genes more frequently than this baseline value, with HIV-1 and SIV displaying the greatest levels of gene targeting (Table 2 and Additional file 3: Table S1). PFV and MMTV by contrast avoided this annotation, accomplishing only about 40% of their integrations within RefSeq genes (*P* = 7.06 × 10^−09^ for PFV and 3.51 × 10^−282^ for MMTV). MMTV also avoided CpG islands (*P* = 9.94 × 10^−21^) and TSSs (*P* = 7.66 × 10^−62^). All other viruses exhibited increased targeting of these genomic features over random, with the gammaretroviruses achieving the greatest levels. Over 40% of PERV integration sites mapped in the vicinity of a CpG island or a TSS (*P* > 2.2 × 10^−308^ for both comparisons to random). The average gene density surrounding MMTV integration sites (8.3 genes per Mb) was lower than the MRC value of 9.2 (*P* > 2.2 × 10^−308^) while all other viruses on average landed in regions that contained more genes per Mb than random. The average gene densities selected by HTLV-1 and PFV, 10.7 and 10.8 genes/Mb, respectively, were only marginally greater than random (*P* = 3.33 × 10^−40^ and 2.95 × 10^−24^, respectively), while the 23.4 genes/Mb value selected by PERV was the greatest among the viruses analyzed (*P* > 2.2 × 10^−308^). As expected, the ability for PFV IN to target chromatin-specific features during integration was decreased significantly when the reaction was conducted with deproteinized cellular tDNA *in vitro* (Table 2; *P* values tabulated in Additional file 4: Figure S3).

Rev-A integrated within RefSeq genes at a frequency of 55.2%, which was significantly more frequent than the MRC value of 45.7% yet less frequent than the HIV-1 value of 74.6% (Table 2 and Additional file 5: Figure S4; *P* = 5.22 × 10^−8^ and 9.08 × 10^−34^, respectively). Notably, this frequency was not significantly different from the other analyzed gammaretroviruses (*P* value range of 0.14 for MoMLV to 0.67 for PERV). The frequencies at which Rev-A and MoMLV targeted CpG islands were also statistically indistinguishable (25.9% vs. 28.4%, respectively; *P* = 0.12), yet were significantly different from the MRC value of 4.6% as well as the HIV-1 frequency of 5.6% (Table 2, Additional file 5: Figure S4). Approximately 25.9% of Rev-A integrations occurred within the 5 kb windows surrounding TSSs, a result that was again statistically indistinguishable from the MoMLV value of 27.8% (*P* = 0.23). The frequencies at which XMRV and PERV targeted CpG islands and TSSs were unique among the viruses studied, including both MoMLV and Rev-A (Table 2, Additional file 5: Figure S4). Rev-A, MoMLV, and XMRV integrated into similarly gene-dense regions of chromatin, whereas the 23.4 gene/Mb value displayed by PERV was more similar to the 19.9 gene/Mb value exhibited by HIV-1 (Table 2, Additional file 5: Figure S4).

### Rev-A IN interacts with and is catalytically stimulated by the BRD4 ET domain *in vitro*

The interaction between MoMLV IN and BET proteins (BRD2-4) in large part determines the promoter-proximal integration profile of this virus [27-29]. The amino acid sequence of the IN C-terminal region, WxϕxxpxxPLbϕbϕR (x, non-conserved position; ϕ, small hydrophobic; p, small polar; b, basic), which dictates binding to BET proteins [29,69,70], is a conserved feature of gammaretroviral IN proteins including Rev-A IN [18]. The BET proteins comprise well-characterized bromodomain (BD) I and II and the ET domain, the latter of which accounts for IN binding (Figure 6A) [27-29,68]. To test if BET proteins interact with Rev-A IN, we expressed and purified hexahistidine-tagged IN and the C-terminal fragment of BRD4_462–720_ that contains the ET domain (Figure 6A). As a control, we substituted glutamic acid for conserved residue Leu-630; the analogous L662E amino acid substitution in BRD2 negated binding to both MoMLV and feline leukemia virus IN proteins *in vitro* [28].

Utilizing a nickel-nitrilotriacetic acid (Ni-NTA) pull down format, Rev-A IN recovered input BRD4_462–720_ protein from the solution but failed to bind detectable levels of BRD4_462–720/L630E_ (Figure 6B). The BET proteins as well as their isolated ET domains can stimulate the concerted integration activity of MoMLV IN *in vitro* [27,28,70]. We accordingly assessed the ability of Rev-A IN to insert oligonucleotide vDNA substrates into supercoiled plasmid tDNA in the presence of BRD4_462–720_ or BRD4_462–720/L630E_. Two major types of integration products were expected under these reaction conditions [1,2,46,66]: the integration of one vDNA end molecule into one strand of plasmid DNA yields a tagged circle that co-migrates with nicked plasmid molecules in an agarose gel whereas the concerted integration of two vDNA ends yields a population of products that migrate as linearized plasmid molecules. As expected [66], Rev-A IN catalyzed a low level of single vDNA end and concerted integration activity in the absence of added BET protein (Figure 7A, compare lane 2 to lane 1). BRD4_462–720_ stimulated IN concerted integration activity in a dose dependent manner, with an approximate fivefold boost in catalysis in the presence of 0.5 μM protein (Figure 7B; *P* < 0.01). On the contrary BRD4_462–720/L630E_ did not appreciably stimulate Rev-A IN activity, even at the highest concentration tested (Figure 7).

## Discussion

Different aspects of the nuclear environment, from global chromatin structure to local tDNA sequence, can influence where retroviruses integrate [18]. Prior work indicated that the forces that dictate these phenotypes may very well operate in an independent fashion. As example, the consensus palindromic sequence at sites of HIV-1 integration was unchanged in cells knocked out for expression of the dominant chromatin targeting factor LEDGF/p75 [24,25]. In this report we have expanded the analysis of local tDNA site determinants across a representative sampling of Retroviridae.

### Central flexibility is a conserved feature of retroviral integration sites

Dinucleotide flexibility is arguably the most crucial sequence-dependent determinant of DNA conformation, with results of numerous studies confirming the specific role of YR steps in DNA bending [71-74]. Base stacking interactions play an even greater role in enforcing the conformation of the DNA double helix than Watson-Crick base pairing or phosphodiester backbone integrity [75-78]. Due to their relative lack of base overlap, YR dinucleotide steps possess the greatest level of inherent flexibility of the four purine/pyrimidine dinucleotides [32]. On the contrary, RY steps share the greatest surface area (RR and YY steps exhibit intermediate base overlap) and consequently display significantly lower propensities for roll, twist, slide, and other DNA bendability characteristics [71-73]. YR steps have accordingly been observed with near 50% frequency at sections of severe kinking in histone-wrapped DNA, while RY steps are by far the least represented at about 14% [79]. PFV selects for YR dinucleotides at the center of its integration sites [12] (Figure 1E), which facilitates IN-mediated minor groove compression of tDNA within the TCC to the point of central base pair unstacking. Our subsequent observation that central YR dinucleotides are enriched within HIV-1 integration sites [33] led us to extend the dinucleotide step analysis of integration sites to 10 additional retroviruses.

HIV-1, SIV, MoMLV, and ASLV integration sites were previously examined for physical properties related to DNA flexibility including A-philicity, protein-induced deformability, and bendability [15]. Both A-philicity and protein-induced deformability are based on dinucleotide frequencies. While all four sets of integration sites displayed similar A-philicity profiles, the protein-induced deformability profile was less dramatic for ASLV than for HIV-1, SIV, and MoMLV, which is consistent with the results reported here. Bendability scores – which are based on trinucleotide frequencies [80] – were higher for HIV-1 and SIV than for MoMLV or ASLV. By contrast, our findings indicate that tDNA is likely to undergo severe bending during MoMLV integration. Although this prior work importantly indicated the general bendable nature of retroviral integration sites, the YR/RY step analyses performed here pinpoint salient bendable tDNA phosphodiester bonds that contribute to vDNA integration. Quantitatively comparing levels of enriched flexibility at specific tDNA bonds using trinucleotide-based metrics may prove suboptimal, as only the integration sites of viruses that yield 5 bp TSDs contain a central trinucleotide. Furthermore, trinucleotide parameters as relating to bendability were originally deduced by probabilistic modeling of DNase I digestion data [80], which is a fairly indirect approach for classifying global levels of bendability for runs of nucleotides. To this point we tallied the frequency of all possible trinucleotide combinations falling at the center of the integration sites for the viruses studied here that yield 5 bp TSDs (EIAV, HIV-1, Rev-A, and SIV), and found that A/T-rich trinucleotides (TAA/TTA, ATA/TAT, and AAA/TTT) were on average preferred by all four viruses compared to the MRC (Additional file 6: Figure S5). Because these sequences do not correlate well with DNase I-based trinucleotide flexibility classification [80], we propose that DNA bendability as applied to retroviral integration sites is better judged by YR/RY dinucleotide step analysis.

Based on the overall similar nature of the YR/RY histograms for the integration sites with 4 bp TSDs (Figure 1), we conclude that each of these viral intasomes is likely to accommodate tDNA with a severe central bend, akin to that observed in the PFV TCC crystal structure [12]. The two PFV IN active sites in the TCC are separated by approximately 26.3 Å, and the central dinucleotide accrues ~55° in negative roll to unstack the tDNA to position scissile phosphodiester bonds that are separated by 4 bp at these two positions [12]. The tDNA bend is considerably more extreme than at any position on nucleosomal DNA. A recent structural study revealed ancillary IN-histone and IN-DNA contacts on the sides of the tDNA-binding groove of the PFV intasome that help to lift nucleosomal DNA from the surface of the histone octamer for productive engagement between the IN active sites [39]. This explains why the nature of tDNA base preferences in the immediate vicinity of the integration site is independent of chromatinization (Figures 4 and 5), and also why nucleosomes may dampen the extent of such preferences (Figure 5).

Our prior work with HIV-1 indicated that the central positions of these integration sites are also likely to kink severely [33]. Due to the odd number of intervening bp, two adjacent tDNA dinucleotide steps seemingly collaborate to elicit the bend required for HIV-1 integration. While the other analyzed viruses that yield 5 bp TSDs in general displayed similar YR/RY histograms as HIV-1, our findings indicate that in these cases one of two central steps contributes more significantly to tDNA deformation during integration. As examples, flexibility at dinucleotide bin position +2 was significantly more important for SIV integration, whereas Rev-A selected for flexibility signatures at dinucleotide bin position +1 (Figure 2E-H). Given the lack of tactile TCC structures, the reasons for this apparent asymmetry are unclear and, in the case of SIV, may be a consequence of the relatively limited number of sites analyzed (Table 1).

Our results indicate that viruses that yield 6 bp TSDs likely require less flexibility at the center of their integration sites than do the viruses that cut tDNA with a 4 bp or 5 bp stagger. Why would some retroviruses require less central flexibility than others? Because phosphodiester bonds apposed by 6 bp are separated by 20.4 Å in canonical B-form DNA, it seems possible that significantly less deformation is required to fit chromosomal DNA into the two active sites of a viral intasome that generates a 6 bp TSD. The positions of the IN active sites in viral intasomes that yield 6 bp TSDs could also be further apart than those observed in PFV TCC structure. Furthermore, the extent of chromatin compaction can influence IN activity *in vitro* [81,82]. Viruses that yield 6 bp TSDs might accordingly display decreased affinity for nucleosomes, which are relative hotspots of YR dinucleotide content [68,79]. To assess if viruses such as ASLV that generate 6 bp TSDs display evidence for relatively nucleosome-depleted regions of chromatin during integration, we extended the boundaries of the tDNA sequence logos to encompass 50 nucleotides (Additional file 7: Figure S6A-C). Most of the analyzed integration sites, including those generated by ASLV, HERV-K, and HTLV-1 (Additional file 7: Figure S6C), showed evidence for nucleosome content adjacent to the local regions of TSD. MMTV and PERV by contrast failed to reveal evidence for periodic A/T-enriched peaks emanating outward from the integration sites. Based on this analysis, the ability to integrate into nucleosomal arrays does not seem to track with the size of TSD. We note that a report published after the submission of this paper indicated that MLV and PFV integration can occur in regions of compact chromatin that tend to disfavor HIV-1 and ASLV integration [45].

### IN-tDNA interactions during retroviral integration

The modeling of thymidine for the preferred tDNA cytidine at the point of vDNA joining suggested that the methyl group at the C5 position of thymine may clash with the scissile phosphodiester bond, providing a structural account for why PFV avoids integration at T residues [12]. As each of the analyzed viruses – with the exception of MMTV – prominently disfavored insertion at a T residue (Additional file 1: Figure S1), this aspect of intasome nucleoprotein structure appears nearly universal. We additionally observed a symmetric preference for thymidine/adenosine at either two or three bases exterior to the TSD boundary across the majority of integration sites (Figures 1, 2 and 3, A-D). Through mutagenesis experiments we previously revealed that HIV-1 IN residue Ser119, which is structurally analogous to PFV IN residue Ala188, helps to determine the identity of tDNA bases three positions upstream from the points of vDNA insertion [33]. Neutral, compact amino acids occupy the analogous position of the CCD α2 helix across retroviruses (Additional file 8: Figure S7), and correlating these residues with tDNA sequence preferences suggests that the polarity of the amino acid side chain dictates IN-tDNA interactions. Specifically, non-polar residues alanine and proline dictate preference for thymidine/adenosine at two bases upstream/downstream of the vDNA insertion sites regardless of TSD length, while polar amino acids serine and threonine shift this preference one base further outward from the tDNA cut, to three bases upstream/downstream. Scrutiny of the corresponding YR/RY peaks reinforces that although serine and threonine dictate the T/A preference at the same relative tDNA position, the specifics of the nucleoprotein contacts are not identical. Serine yields the T/A preference at positions −3/+7 for HIV-1, while the S119T IN mutation [33] as well as the threonine that is naturally present in EIAV IN, switches this preference to A/T (Figure 2). Recent studies have determined integration sites of HIV-1 IN mutants that harbor all possible small amino acid substitutions for Ser119 in the CCD (S119A, S119T, S119P, S119G) [33,83]. Consistent with our observations, Ser119 as well as the Thr substituent were modeled to interact with bases at tDNA positions −3/+7 while Ala and Gly were modeled to interact with positions −2/+6 [83].

### Rev-A integration distribution and BET proteins

Our results reveal that Rev-A shares similar integration site distribution patterns with other gammaretroviruses (Table 2). Of the four gammaretroviruses analyzed in this study, the targeting of chromatin-specific features was most similar between Rev-A and MoMLV (Table 2 and Additional file 5: Figure S4). Purified BRD4_462–730_ interacted with Rev-A IN and stimulated its concerted integration activity, and the L630E amino acid substitution in BRD4 counteracted the protein-protein interaction (Figures 6 and 7). Rev-A and MoMLV are thus likely directed to integrate into signature chromatin features using similar BET protein-IN mediated interactions. The unique chromatin targeting preferences of PERV and XMRV suggest that these viruses may interact with additional host factors to guide promoter-proximal integration events.

## Conclusions

The work presented here clarifies that conserved palindromic sequences at sites of retroviral DNA integration reflect the requirement for a central tDNA bend and that the sharpness of the required deformation is reduced for viruses that generate 6 bp TSDs. It seems plausible that retroviruses have convergently evolved to select not necessarily a specific sequence of nucleotides at integration sites, but rather combinations of bases that yield a flexibility pattern that is favorable for tDNA incorporation into the TCC. Retroviral base preferences and associated flexibility profiles appear largely independent of tDNA chromatinization, suggesting that IN interactions with tDNA nucleotides dictate integration site selection on the local scale.

## Methods

### Plasmids, cell culture, and virus infection

A human BRD4 expression construct was purchased from Addgene (plasmid #14441). The region of BRD4 corresponding to amino acids 462–720 was PCR-amplified using primers AE5271 (5′-GATATACCCGGGGAGGAGCCAGTGGTGGCCGTG) and AE5270 (5′-GCAGCACTCGAGTTACTCTGTTTCGGAGTCTTC). The PCR product was cleaved with XhoI and XmaI, and ligated to XhoI/XmaI-digested pCPH6P [84]. The L630E amino acid substitution was introduced by site-directed mutagenesis (Stratagene QuickChange II kit) using primers AE5362 (5′-AAGCTCCCCGGCGAGAAGGAGGGCCGCGTGGTGCACATC) and AE5363 (5′-GATGTGCACCACGCGGCCCTCCTTCTCGCCGGGGAGCTT). Plasmids pCPH6P-RevA-IN [66], pJD215 [85], pSW253 [86], and pCG-VSV-G [25] were previously described.

HEK293T and HeLa cells were cultured in Dulbecco’s Modified Eagle Media supplemented to contain 10% fetal bovine serum, 100 IU/mL penicillin, and 100 μg/mL streptomycin. HEK293T cells were transfected with pJD215, pSW253, and pCG-VSV-G at the ratio of 4.5:4.5:1 using PolyJet (SignaGen). After 48 h, the cell-free supernatant was filtered through 0.45 μm filters, concentrated by ultracentrifugation at 200,000 g for 1 h, and treated with 40 U/mL DNase Turbo (Ambion). Viral titer was determined by infecting 3 x 10^5^ HeLa cells with two-fold dilutions of virus in the presence of 500 μg/mL G418 (Life Technologies) and counting neomycin-resistant colony forming units after 7 d. For integration site sequencing, HEK293T cells (5 x 10^6^) were infected and treated similarly for 7 d to select for transductants and to allow for dissolution of unintegrated vDNA.

### Expression and purification of recombinant proteins

His_6_-tagged Rev-A IN was expressed in *Escherichia coli* and purified essentially as previously described [66], but without hexahistidine tag cleavage. BRD4_462–720_ and BRD4_462–720/L630E_ were expressed in *E. coli* strain PC2 [84] by overnight induction with 1 mM isopropyl-β-D-thiogalactopyranoside at 18°C. The bacterial pellets were dissolved in 50 mM Tris HCl, pH 7.5, 500 mM NaCl, and 1 mM phenylmethanesulfonylfluoride, lysed by sonication, and clarified by centrifugation at 40,000 g for 1 h. BRD4_462–720_ and BRD4_462–720/L630E_ were bound to a HisTrap HP column and eluted using a 20–500 mM imidazole gradient on an AKTA purifier liquid chromatography system (GE Healthcare). Fractions containing the protein of interest were pooled together and quickly diluted 5-fold with 50 mM Tris HCl, pH 7.5 to reduce the concentration of NaCl to 100 mM and then loaded on a HiTrap Heparin column and eluted with a 100–1000 mM NaCl gradient. Finally, the proteins were purified by gel filtration using a Superdex 200 column in 50 mM Tris HCl, pH 7.5, 500 mM NaCl, 2 mM dithiothreitol (DTT). Chromatography columns were purchased from GE Healthcare.

### Pull-down assay

For *in vitro* Ni-NTA pull-down assays, 10 μg of His_6_-tagged Rev-A IN in 100 μL pull-down buffer (25 mM Tris HCl pH 7.5, 150 mM NaCl, 25 μM ZnCl_2_, 0.1% (v/v) Nonidet P40, 20 mM imidazole) was mixed with 10 μL settled volume of Ni-NTA beads (Thermo Scientific) previously washed with pull-down buffer. Following incubation at 4°C for 2 h with gentle agitation, 10 μg BSA and 10 μg of BRD4_462–720_ or BRD4_462–720/L630E_ were added, and mixtures were incubated overnight at 4°C. The beads were washed five times with pull-down buffer and briefly centrifuged for 1 min at 1,300 g, and were then resuspended in 20 μL 2X sodium dodecyl sulfate (SDS) gel loading buffer and boiled for 10 min. The resulting supernatant was analyzed by denaturing gel electrophoresis on a 10% acrylamide gel. Proteins were detected by staining with Coomassie blue.

### DNA strand transfer activity assay

The *in vitro* concerted integration assays were carried out as previously for described Rev-A IN following removal of the His_6_-tag by site-specific proteolysis [66]. Briefly, 1 μM Rev-A IN mixed with 0.5 μM vDNA and 4 nM pGEM-3 tDNA, in 40 μL of 20 mM HEPES pH 7.4, 50 mM NaCl, 5 mM MgCl_2_, 4 μM ZnCl_2_, and 10 mM DTT, was incubated for 1 h at 37°C with varying concentrations of BRD4_462–720_ or BRD4_462–720/L630E_ (0.05/0.15/0.35/0.5 μM). Reactions were stopped by adding 25 mM EDTA and 0.5% SDS, and deproteinized by digestion with proteinase K. Products were precipitated with ethanol and analyzed by electrophoresis through 1.5% agarose gels. DNA was detected by staining with ethidium bromide. Concerted integration products were measured by band intensity quantification using the Molecular Imager® Gel Doc TM XR+ System with Image Lab software (Bio-Rad).

### Sequencing of Rev-A integration sites

Genomic DNA (20 μg) isolated using the DNeasy Blood and Tissue Kit (Qiagen) was digested overnight with AvrII, NheI, and SpeI and purified using the QIAquick PCR Purification Kit (Qiagen). A double-stranded asymmetric linker was prepared by annealing 10 μM of oligonucleotides AE5237 (5′-[Phosp]CTAGGCAGCCCG[AmC7-Q]-3′) and AE5238 (GTAATACGACTCACTATAGGGCACGCGTGGTCGACGGCCCGGGCTGC) by heating to 90°C in 10 mM Tris–HCl, pH 8.0 and 0.1 mM EDTA and slowly cooling to room temperature. Linker DNA (1.5 μM) was ligated with digested cellular DNA (1 μg) overnight at 16°C in four parallel reactions, and the DNAs were pooled and re-purified using the QIAquick PCR Purification Kit. Nested PCR was used to selectively amplify integration sites, with reactions multiplexed into eight separate samples per PCR stage. First- and second-round linker primers were AE5240 (5′-GACTCACTATAGGGCACGCGT) and AE5242 (5′-GTCGACGGCCCGGGCTGCCTA), and first- and second round Rev-A U5 primers were AE6121 (5′-GCAGGGATCCGGACTG) and AE6122 (5′-CCGTAGTACTTCGGTACAAC), respectively. PCRs were incubated at 94°C for 2 min, followed by 30 cycles at 94°C for 15 sec, 55°C for 30 sec, and 68°C for 45 sec, which was followed by a final extension for 10 min at 68°C. Pooled PCRs were purified using the QIAquick PCR Purification Kit, and standard Illumina adapters were ligated onto the amplicons prior to sequencing on the Illumina MiSeq platform at the Data-Farber Cancer Institute Molecular Biology Core Facilities. Sequences were mapped to hg19 version of human genome using BLAT, ensuring that the genomic match starts immediately after TACTTCGGTACAACA sequence, which corresponds to the processed Rev-A U5 end. Bioinformatics analysis of Rev-A integration sites was performed as described previously [87].

### Statistical analysis

An MRC dataset of 282,824 sites was created by selecting random genomic positions in proximity (<500 bp) of a AvrII, NheI or SpeI recognition site. The sequence immediately abutting each random site, truncated to 97 bp or less (to simulate Illumina read length), was subjected to the genomic alignment procedure described above. Differences in nucleotide sequence from random among retroviral integration sites were determined by chi-square analysis relative to the above random control. Statistical differences with respect to YR/RY frequency plots and genomic distribution of integration sites was calculated by Fisher’s Exact Test using *R* [88].

## Additional files

Additional file 1: Figure S1. Nucleotide preferences at tDNA base positions surrounding retroviral integration sites. The number in parentheses indicates the number of unique integration sites analyzed for each virus. Chi-square analysis was used to calculate *P* values by comparing observed nucleotide frequencies to the expected frequency based on 282,824 random points in the human genome. Significant differences are marked by blue shade and bold character (*P* < 0.05). Nucleotide frequencies that differed from the neutral value of 100% by more than 40 are highlighted in red (<60%) and green (>140%). Additional file 2: Figure S2. Statistical analysis of YR/RY dinucleotide frequencies across retroviral integration sites. (A and B) *P* values calculated by Fisher’s Exact Test for statistical comparison of YR and RY dinucleotide frequency profiles as compared to the MRC of 282,824 randomly-generated sites for viruses that yield 4 bp TSDs. (C and D) Statistical analysis of dinucleotide frequencies for viruses that yield 5 bp TSDs. (E and F) Analysis of dinucleotide frequency statistics for viruses with 6 bp TSDs. The dotted gray horizontal line in all panels demarcates the statistical cutoff value of 1.3 (−log_10_(0.05)). Curve flattening results from the statistical cutoff of 2.2 × 10^−308^ of the utilized statistical package [88]. Asterisks denote curves graphed on the secondary y-axis to the right of the charts. Additional file 3: Table S1.
*P* values for comparison of retroviral integration site distributions versus MRC. Values > 0.05 are highlighted in bold italics. Additional file 4: Figure S3.
*P* values for comparison of PFV integration site distribution in deproteinized tDNA and from virus-infected cells to the MRC dataset. Counts of integration sites within RefSeq genes and relative to CpG islands and TSSs, as well as regional gene densities, are listed in Table 2. Additional file 5: Figure S4.
*P* values for comparison of Rev-A integration site distribution to other gammaretroviruses, HIV-1, PFV, and to the MRC dataset. Numbers of integration sites within RefSeq genes and nearby CpG islands and TSSs, as well as regional gene density profiles, are listed in Table 2. Additional file 6: Figure S5. Trinucleotide frequencies at centers of 5 bp TSDs. The frequency of all possible trinucleotides residing at the central three bases of EIAV, HIV-1, Rev-A, and SIV integration sites was computed and compared to the random distribution given by the MRC dataset. Y-axis values represent the percent increase in usage over random. The x-axis displays the most frequently-utilized trinucleotides arranged from left to right. Additional file 7: Figure S6. Extended sequence logos depicting base preferences at a total of 50 bases surrounding retroviral integration sites. The Y-axis scales were set to 0.2 bits for all logos in order to highlight T/A periodicity, and thus some of the overly prominent base preferences at the centers of the integration sites are slightly obscured. (A) Sequence logos for viruses that yield 4 bp TSDs are compared to the average sequence of chicken erythrocyte nucleosomal DNA and to the *in vitro* dataset of recombinant PFV integration sites. (B) Same as in panel A, except the analyzed viruses generate 5 bp TSDs. The *in vitro* HIV-1 integration dataset is from ref. [33]. (C) Extended sequence logos for viruses that yield 6 bp TSDs, compared to the average nucleosome content of chicken DNA. Additional file 8: Figure S7. Amino acid sequence alignment of CCD α2 helix residues for the viruses analyzed in this study. The location of secondary structural elements has been documented crystallographically for HIV-1 [89], ASLV [90], SIV [91], and PFV [2] INs. Residues analogous to Ala188 in PFV and Ser119 in HIV-1 INs are highlighted in yellow. The active site residue that is analogous to Asp185 in PFV IN and Asp116 in HIV-1 IN is in red type.

## Abbreviations

IN: Integrase
vDNA: Viral DNA
TCC: Target capture complex
tDNA: Target DNA
TSD: Target site duplication
TSS: Transcriptional start site
LEDGF: Lens epithelium-derived growth factor
BET: Bromodomain and extraterminal domain
MoMLV: Moloney murine leukemia virus
MMTV: Mouse mammary tumor virus
PFV: Prototype foamy virus
CCD: Catalytic core domain
Y: Pyrimidine
R: Purine
HIV-1: Human immunodeficiency virus type 1
Rev-A: Reticuloendotheliosis virus strain A
ET: Extraterminal
PERV: Porcine endogenous retrovirus
XMRV: Xenotropic murine leukemia virus-related virus
ASLV: Avian sarcoma-leukosis virus
HTLV-1: Human T-lymphotropic virus type 1
HERV-K: Human endogenous retrovirus K family
SIV: Simian immunodeficiency virus
EIAV: Equine infectious anemia virus
MRC: Matched random control
hg19: Human genome build 19
BD: Bromodomain
Ni-NTA: Nickel-nitrilotriacetic acid
DTT: Dithiothreitol
SDS: Sodium dodecyl sulfate
