# Supplementary material for: Key determinants of target DNA recognition by retroviral intasomes
Source: Retrovirology. 2015 Apr 30;12:39. doi: 10.1186/s12977-015-0167-3 (PMC4422553; doi:10.1186/s12977-015-0167-3)
Supplement: Additional file 1: Figure S1. — Nucleotide preferences at tDNA base positions surrounding retroviral integration sites. The number in parentheses indicates the number of unique integration sites analyzed for each virus. Chi-square analysis was used to calculate P values by comparing observed nucleotide frequencies to the expected frequency based on 282,824 random points in the human genome. Significant differences are marked by blue shade and bold character (P < 0.05). Nucleotide frequencies that differed from the neutral value of 100% by more than 40 are highlighted in red (<60%) and green (>140%). [file 12977_2015_167_MOESM1_ESM.pdf]

PFV (2,924)*	R	G	G	D	G	S	Y	R	S	C	A/H	C	C	H	C
	-5	-4	-3	-2	-1	0	1	2	3	4	5	6	7	8	9
A	119	87	100	111	75	85	78	124	31	78	132	93	84	91	82
C	72	102	74	50	82	165	131	70	157	177	92	130	145	109	141
G	119	146	137	94	187	159	70	128	169	81	54	74	103	75	102
T	87	79	93	129	81	28	120	78	83	82	106	103	81	121	89
#-log(*P*)	23	39	27	50	116	200	40	42	201	95	46	20	38	18	30

MoMLV (53,463)	R	G	S	T/W	G/S	S/V	T	V	S/B	C	A	G/V	C/B	B	B
	-5	-4	-3	-2	-1	0	1	2	3	4	5	6	7	8	9
A	112	98	82	80	80	110	77	119	59	75	178	101	79	79	85
C	93	102	130	49	118	136	93	98	120	137	58	107	132	120	118
G	118	123	126	31	132	146	84	110	128	123	61	123	106	96	104
T	81	84	79	205	87	31	139	76	109	83	79	78	93	111	100
#-log(*P*)	257	211	>301	>301	>301	>301	>301	>301	>301	>301	>301	301	>301	294	160

PERV (1,668)	G/V	G/S	S	T/W	G	G/V	T/Y	A	C/B	C/S	A/W	S	C/S	C/B	C/S
	-5	-4	-3	-2	-1	0	1	2	3	4	5	6	7	8	9
A	93	87	46	95	48	72	75	132	35	56	189	53	66	73	71
C	116	109	184	34	69	127	104	88	198	273	51	150	160	129	150
G	136	146	158	46	283	212	80	98	125	79	34	193	107	114	116
T	72	74	54	189	54	30	135	78	81	39	91	48	84	98	83
#-log(*P*)	19	24	130	137	295	157	21	16	123	282	134	134	41	15	31

XMRV (5,487)	N	N	G	T/W	S	C/V	T	A/R	G/B	C/S	A	C	C	Y	C
	-5	-4	-3	-2	-1	0	1	2	3	4	5	6	7	8	9
A	113	105	92	74	76	115	68	143	38	75	196	93	87	84	84
C	95	97	111	47	133	153	88	77	123	142	41	124	119	111	119
G	109	113	128	30	131	124	66	104	142	127	55	105	97	89	103
T	84	88	81	213	82	31	164	71	118	77	77	87	101	116	101
#-log(*P*)	17	10	36	>301	81	257	209	105	203	101	>301	22	14	22	18

EIAV (1,172)	A	T	A/W	D	G/R	G/V	T/W	A/W	A/W	C/B	C/Y	A/H	T/W	H	T/H
	-5	-4	-3	-2	-1	0	1	2	3	4	5	6	7	8	9
A	129	108	191	104	116	99	107	140	158	46	76	121	113	109	95
C	76	77	21	79	75	127	52	46	51	155	136	104	37	100	98
G	80	89	40	105	132	153	49	54	47	125	84	75	21	72	68
T	102	116	108	108	80	45	162	131	111	100	110	94	188	110	130
#-log(*P*)	10	5.2	112	2.6	13	40	53	44	52	41	12	6.3	113	5.0	11

HIV-1 (335,968)	N	N	T/K	D	G	G/V	T/W	W	A/W	C/B	C	H	A/M	V	N
	-5	-4	-3	-2	-1	0	1	2	3	4	5	6	7	8	9
A	104	83	70	100	93	85	92	119	149	33	76	118	163	103	99
C	96	113	62	50	75	113	68	76	67	201	172	128	99	101	106
G	102	96	99	126	176	205	68	78	71	119	78	51	64	118	93
T	98	111	156	118	74	31	152	114	92	85	89	97	65	82	102
#-log(*P*)	71	>301	>301	>301	>301	>301	>301	>301	>301	>301	>301	>301	>301	>301	138

REV-A (834)	C/V	A/V	S/V	T/H	G/V	G/V	T	N	V	C/B	C	A/D	S	C/B	C
	-5	-4	-3	-2	-1	0	1	2	3	4	5	6	7	8	9
A	93	128	105	83	102	88	80	101	115	50	90	147	69	81	85
C	148	101	132	115	98	127	96	95	101	167	137	65	137	122	136
G	103	111	124	47	159	164	92	115	100	129	107	86	130	102	99
T	74	64	57	145	61	48	128	92	84	83	80	87	82	103	92
#-log(*P*)	12	11	15	22	20	32	6.1	0.9	2.0	34	7.4	17	15	3.3	5.6

SIV (168)	C	A/D	G/V	T/W	G/R	G/V	T/H	T/D	A/D	C/Y	C/H	A/W	C/B	T/H	A
	-5	-4	-3	-2	-1	0	1	2	3	4	5	6	7	8	9
A	86	140	75	119	89	87	118	101	160	46	63	168	48	86	122
C	120	43	66	23	53	83	68	40	38	257	241	38	214	106	94
G	114	86	254	50	232	225	35	72	64	55	26	32	68	68	86
T	91	112	44	170	58	37	149	161	106	78	89	122	91	134	92
#-log(*P*)	0.5	3.9	22	11	15	16	6.1	6.7	7.1	23	19	11.0	12.9	1.8	0.5
	N	S	T	N	G	S/V	T	B	V	B	S	N	V	A	S

Additional File 1: Figure S1 (page 1 of 2)

ASLV (916)	-5	-4	-3	-2	-1	0	1	2	3	4	5	6	7	8	9
A	101	78	80	109	107	71	89	84	106	89	63	93	115	**144**	85
C	88	125	63	95	77	**174**	91	109	102	101	**144**	106	106	71	121
G	124	124	79	106	129	**153**	83	100	107	111	138	86	105	78	120
T	90	86	**159**	91	90	**40**	128	109	88	103	80	112	78	90	87
#-log(*P*)	**3.0**	**8.1**	**30**	0.8	**5.7**	**59**	**6.2**	**2.0**	1.0	1.0	**23**	**1.7**	**3.7**	**17**	**5.2**

	R	N	R	T	N	S	T	H	N	B	C/S	V	A	C/B	Y
HERV-K (1,071)	-5	-4	-3	-2	-1	0	1	2	3	4	5	6	7	8	9
A	121	104	109	73	90	84	92	97	102	88	68	113	132	68	89
C	89	90	80	90	105	130	99	106	100	102	**140**	108	89	139	125
G	112	108	132	67	120	135	78	79	101	107	128	101	95	97	81
T	79	98	84	**158**	93	70	124	114	97	106	85	81	80	108	107
#-log(*P*)	**6.6**	0.6	**8.4**	**33**	**2.4**	**17**	**6.1**	**3.2**	0.0	1.2	**19**	**3.3**	**9.4**	**14**	**5.0**

	A	R	G/V	T/H	G/V	G/V	R	W	A	B	C/B	C/B	A/W	C/B	Y
HTLV-1 (6,820)	-5	-4	-3	-2	-1	0	1	2	3	4	5	6	7	8	9
A	130	116	115	78	95	101	117	109	132	84	61	70	**196**	**38**	85
C	85	75	101	61	112	110	89	72	77	114	**146**	**142**	**41**	**174**	130
G	96	118	**171**	**36**	**145**	**154**	119	75	76	93	110	115	**59**	116	76
T	83	90	**37**	**195**	68	**54**	78	128	99	112	100	90	78	101	112
#-log(*P*)	**57**	**42**	**>301**	**>301**	**107**	**182**	**46**	**78**	**78**	**24**	**132**	**102**	**>301**	**>301**	**59**

	N	N	N	Y	N	N	T	N	N	N	C	C	N	Y	T
MMTV (178,574)	-5	-4	-3	-2	-1	0	1	2	3	4	5	6	7	8	9
A	101	99	106	87	91	91	91	88	91	94	77	83	105	77	90
C	102	96	88	105	106	114	94	103	106	110	127	127	102	122	99
G	102	97	113	88	113	113	92	94	103	98	109	101	91	80	89
T	96	106	94	118	97	90	119	113	103	101	98	98	100	123	119
#-log(*P*)	**30**	**58**	**>301**	**>301**	**262**	**>301**	**>301**	**>301**	**135**	**131**	**>301**	**>301**	**95**	**>301**	**>301**

(green)	>140%
(red)	<60%
**bold**	***P* value vs random <0.05 = -log10(*P*) >1.301**
*	Number of unique samples per virus

Additional File 1: Figure S1 (page 2 of 2)
