# Supplementary figures and images for: Key determinants of target DNA recognition by retroviral intasomes
Source: Retrovirology. 2015 Apr 30;12:39. doi: 10.1186/s12977-015-0167-3 (PMC4422553; doi:10.1186/s12977-015-0167-3)

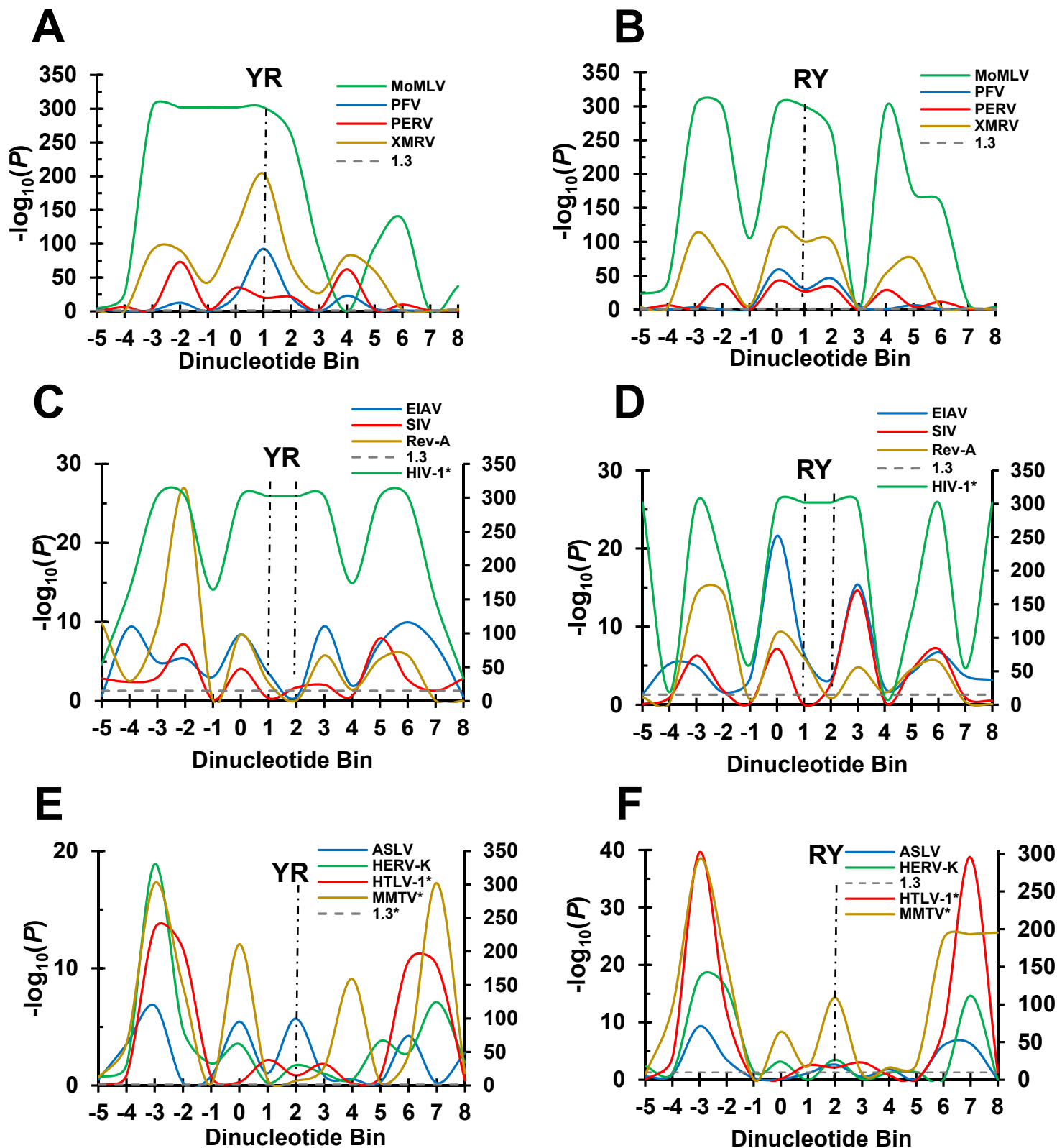

**Additional File 2: FIGURE S2**

Supplement: Additional file 2: Figure S2. — Statistical analysis of YR/RY dinucleotide frequencies across retroviral integration sites. (A and B) P values calculated by Fisher’s Exact Test for statistical comparison of YR and RY dinucleotide frequency profiles as compared to the MRC of 282,824 randomly-generated sites for viruses that yield 4 bp TSDs. (C and D) Statistical analysis of dinucleotide frequencies for viruses that yield 5 bp TSDs. (E and F) Analysis of dinucleotide frequency statistics for viruses with 6 bp TSDs. The dotted gray horizontal line in all panels demarcates the statistical cutoff value of 1.3 (−log10(0.05)). Curve flattening results from the statistical cutoff of 2.2 × 10−308 of the utilized statistical package [88]. Asterisks denote curves graphed on the secondary y-axis to the right of the charts. [file 12977_2015_167_MOESM2_ESM.pdf]

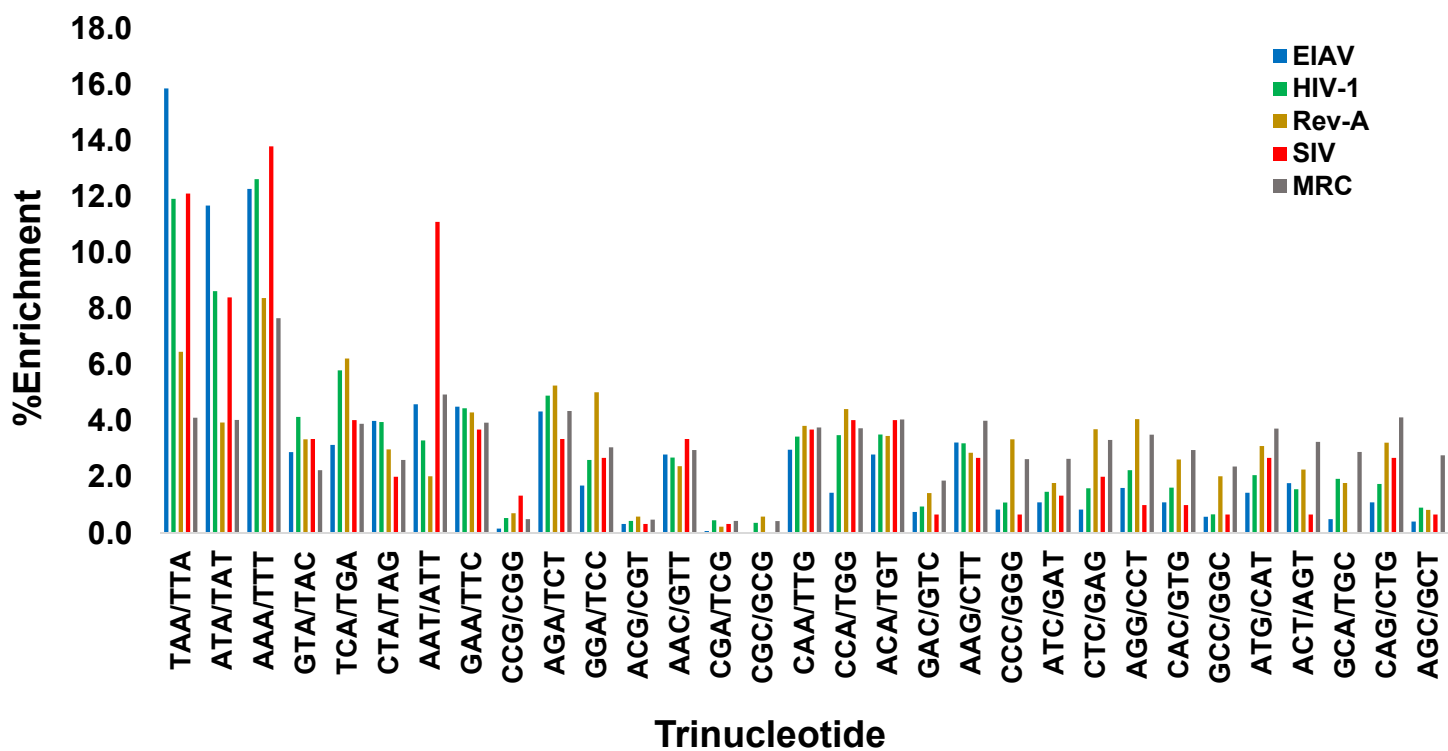

**Additional File 6: FIGURE S5**

Supplement: Additional file 6: Figure S5. — Trinucleotide frequencies at centers of 5 bp TSDs. The frequency of all possible trinucleotides residing at the central three bases of EIAV, HIV-1, Rev-A, and SIV integration sites was computed and compared to the random distribution given by the MRC dataset. Y-axis values represent the percent increase in usage over random. The x-axis displays the most frequently-utilized trinucleotides arranged from left to right. [file 12977_2015_167_MOESM6_ESM.pdf]
