# Supplementary material for: Key determinants of target DNA recognition by retroviral intasomes
Source: Retrovirology. 2015 Apr 30;12:39. doi: 10.1186/s12977-015-0167-3 (PMC4422553; doi:10.1186/s12977-015-0167-3)
Supplement: Additional file 3: Table S1. — P values for comparison of retroviral integration site distributions versus MRC. Values > 0.05 are highlighted in bold italics. [file 12977_2015_167_MOESM3_ESM.pdf]

Table S1 *P* values for comparison of retroviral integration site distributions versus MRC

Library	Within Refseq genes	Within 5 kb (+/- 2.5 kb) of CpG (%)	Within 5 kb (+/- 2.5 kb) of TSS (%)	Average gene density per Mb (+/- 0.5 Mb) of integration sites
PFV	7.06E-09	1.16E-108	5.88E-85	2.95E-24
PFV (*in vitro*)	2.81E-07	1.73E-23	***0.077***	1.60E-60
MoMLV	>2.2E-308	>2.2E-308	>2.2E-308	>2.2E-308
PERV	2.23E-17	>2.2E-308	>2.2E-308	>2.2E-308
XMRV	5.84E-54	2.54E-307	>2.2E-308	>2.2E-308
EIAV	3.62E-19	0.025	***0.137***	4.49E-25
HIV-1	>2.2E-308	3.39E-78	3.37E-51	>2.2E-308
Rev-A	8.98E-40	***0.675***	***0.591***	4.68E-59
SIV	5.22E-08	1.65E-96	2.12E-90	3.94E-77
ASLV	5.05E-11	1.65E-96	***0.939***	1.62E-09
HERV-K	1.72E-03	9.84E-24	5.73E-12	5.53E-112
HTLV-1	***0.468***	1.49E-28	8.10E-12	3.33E-40
MMTV	3.51E-282	9.94E-21	7.66E-62	>2.2E-308

Additional File 3: TABLE S1
