# Supplementary material for: Key determinants of target DNA recognition by retroviral intasomes
Source: Retrovirology. 2015 Apr 30;12:39. doi: 10.1186/s12977-015-0167-3 (PMC4422553; doi:10.1186/s12977-015-0167-3)
Supplement: Additional file 4: Figure S3. — P values for comparison of PFV integration site distribution in deproteinized tDNA and from virus-infected cells to the MRC dataset. Counts of integration sites within RefSeq genes and relative to CpG islands and TSSs, as well as regional gene densities, are listed in Table 2. [file 12977_2015_167_MOESM4_ESM.pdf]

Within RefSeq Gene

	Infection	In Vitro
**MRC**	7.06×10^{-09}	2.81×10^{-07}
**Infection**		3.20×10^{-13}

± 2.5 kB of a CpG Island

	Infection	In Vitro
**MRC**	1.16×10^{-108}	1.73×10^{-23}
**Infection**		2.37×10^{-60}

± 2.5 kB of a TSS

	In Vitro	In Vitro
**MRC**	5.88×10^{-85}	***0.08***
**Infection**		5.16×10^{-67}

Avg. Genes/Mb

	Infection	In Vitro
**MRC**	2.95×10^{-24}	1.60×10^{-60}
**Infection**		2.61×10^{-04}

Additional File 4: FIGURE S3
