# Supplementary material for: Key determinants of target DNA recognition by retroviral intasomes
Source: Retrovirology. 2015 Apr 30;12:39. doi: 10.1186/s12977-015-0167-3 (PMC4422553; doi:10.1186/s12977-015-0167-3)
Supplement: Additional file 5: Figure S4. — P values for comparison of Rev-A integration site distribution to other gammaretroviruses, HIV-1, PFV, and to the MRC dataset. Numbers of integration sites within RefSeq genes and nearby CpG islands and TSSs, as well as regional gene density profiles, are listed in Table 2. [file 12977_2015_167_MOESM5_ESM.pdf]

Within RefSeq Gene

	Rev-A	MoMLV	XMRV	PERV	PFV	HIV-1
**MRC**	5.22 x 10^{-08}	<2.2 x 10^{-308}	5.84 x 10^{-54}	2.23 x 10^{-17}	7.06 x 10^{-09}	<2.2 x 10^{-308}
	**Rev-A**	***0.14***	***0.57***	***0.67***	***4.47 x 10^{-14}***	***9.08 x 10^{-34}***
		**MoMLV**	0.03	0.19	2.92 x 10^{-75}	<2.2 x 10^{-308}
			**XMRV**	0.93	7.17 x 10^{-44}	7.24 x 10^{-187}
				**PERV**	6.89 x 10^{-25}	1.24 x 10^{-59}
					**PFV**	<2.2 x 10^{-308}

± 2.5 kB of a CpG Island

	Rev-A	MoMLV	XMRV	PERV	PFV	HIV-1
**MRC**	1.65 x 10^{-96}	<2.2 x 10^{-308}	2.54 x 10^{-307}	<2.2 x 10^{-308}	1.16 x 10^{-108}	3.39 x 10^{-78}
	**Rev-A**	***0.12***	***1.68 x 10^{-06}***	***1.98 x 10^{-28}***	***1.26 x 10^{-11}***	***3.05 x 10^{-80}***
		**MoMLV**	4.89 x 10^{-57}	4.67 x 10^{-66}	2.07 x 10^{-58}	<2.2 x 10^{-308}
			**XMRV**	1.03 x 10^{-122}	1.86 x 10^{-04}	<2.2 x 10^{-308}
				**PERV**	4.16 x 10^{-127}	<2.2 x 10^{-308}
					**PFV**	8.56 x 10^{-81}

± 2.5 kB of a TSS

	Rev-A	MoMLV	XMRV	PERV	PFV	HIV-1
**MRC**	2.12 x 10^{-90}	<2.2 x 10^{-308}	<2.2 x 10^{-308}	<2.2 x 10^{-308}	5.88 x 10^{-85}	3.37 x 10-^{51}
	**Rev-A**	***0.23***	***1.4 x 10^{-04}***	***3.31 x 10^{-13}***	***1.26 x 10^{-13}***	***1.09 x 10^{-104}***
		**MoMLV**	9.43 x 10^{-37}	4.54 x 10^{-28}	1.86 x 10^{-62}	<2.2 x 10^{-308}
			**XMRV**	3.01 x 10^{-60}	2.79 x 10^{-10}	<2.2 x 10^{-308}
				**PERV**	6.24 x 10^{-85}	<2.2 x 10^{-308}
					**PFV**	2.78 x 10^{-108}

Avg. Genes/Mb

	Rev-A	MoMLV	XMRV	PERV	PFV	HIV-1
**MRC**	1.24 x 10^{-06}	<2.2 x 10^{-308}	8.53 x 10^{-76}	0.41	1.61 x 10^{-06}	5.42 x 10^{-13}
	**Rev-A**	***0.03***	***0.03***	***9.39 x 10^{-06}***	***0.05***	***1.61 x 10^{-05}***
		**MoMLV**	0.92	1.01 x 10^{-25}	3.35 x 10^{-16}	<2.2 x 10^{-308}
			**XMRV**	3.17 x 10^{-21}	5.85 x 10^{-12}	4.46 x 10^{-65}
				**PERV**	4.0 x 10^{-04}	0.11
					**PFV**	1.4 x 10^{-04}

Additional File 5: FIGURE S4
