# Supplementary material for: Key determinants of target DNA recognition by retroviral intasomes
Source: Retrovirology. 2015 Apr 30;12:39. doi: 10.1186/s12977-015-0167-3 (PMC4422553; doi:10.1186/s12977-015-0167-3)
Supplement: Additional file 7: Figure S6. — Extended sequence logos depicting base preferences at a total of 50 bases surrounding retroviral integration sites. The Y-axis scales were set to 0.2 bits for all logos in order to highlight T/A periodicity, and thus some of the overly prominent base preferences at the centers of the integration sites are slightly obscured. (A) Sequence logos for viruses that yield 4 bp TSDs are compared to the average sequence of chicken erythrocyte nucleosomal DNA and to the in vitro dataset of recombinant PFV integration sites. (B) Same as in panel A, except the analyzed viruses generate 5 bp TSDs. The in vitro HIV-1 integration dataset is from ref. [33]. (C) Extended sequence logos for viruses that yield 6 bp TSDs, compared to the average nucleosome content of chicken DNA. [file 12977_2015_167_MOESM7_ESM.pdf]

A

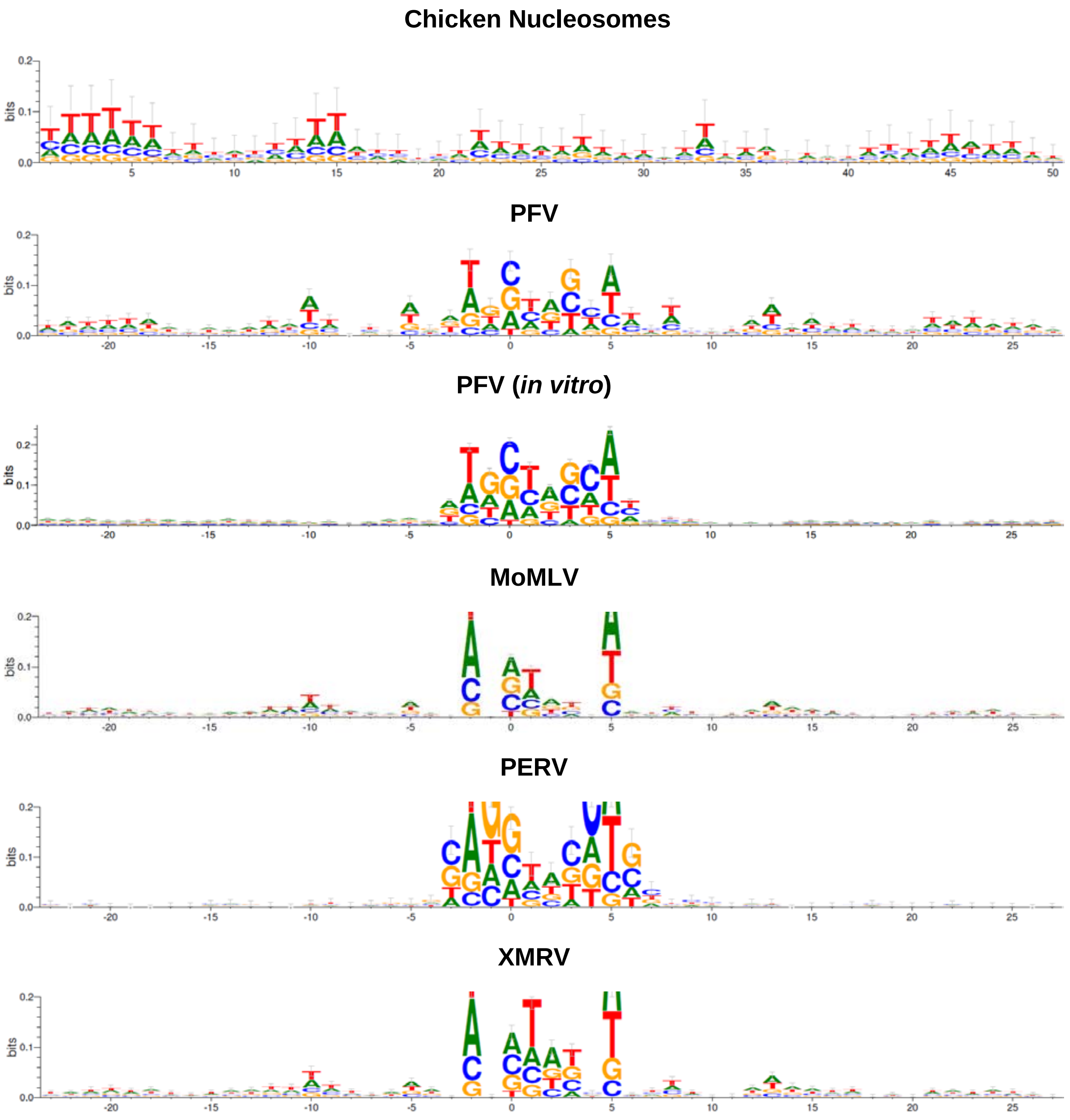

Additional File 7: FIGURE S6 (page 1 of 3)

B

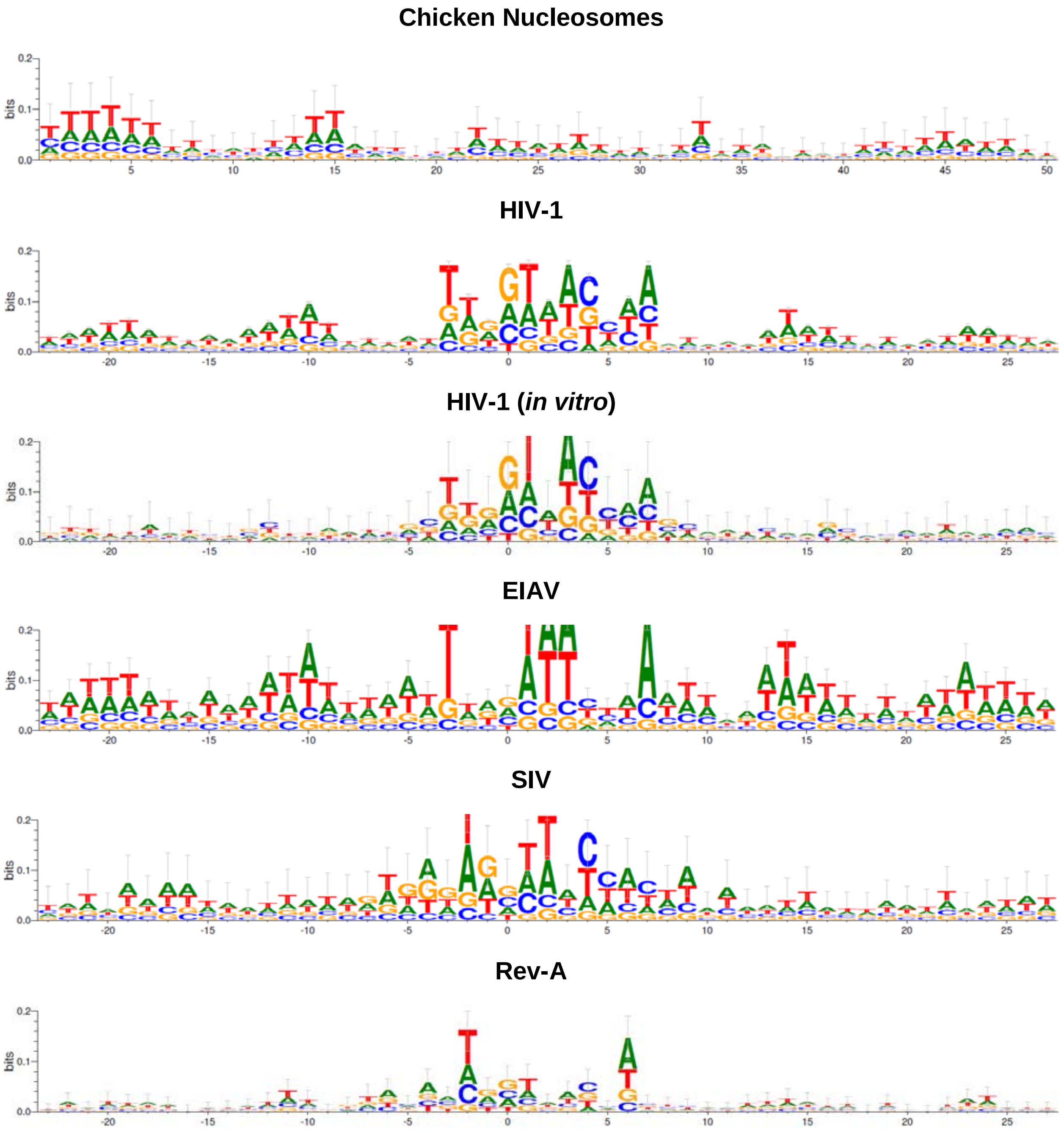

Additional File 7: FIGURE S6 (page 2 of 3)

C

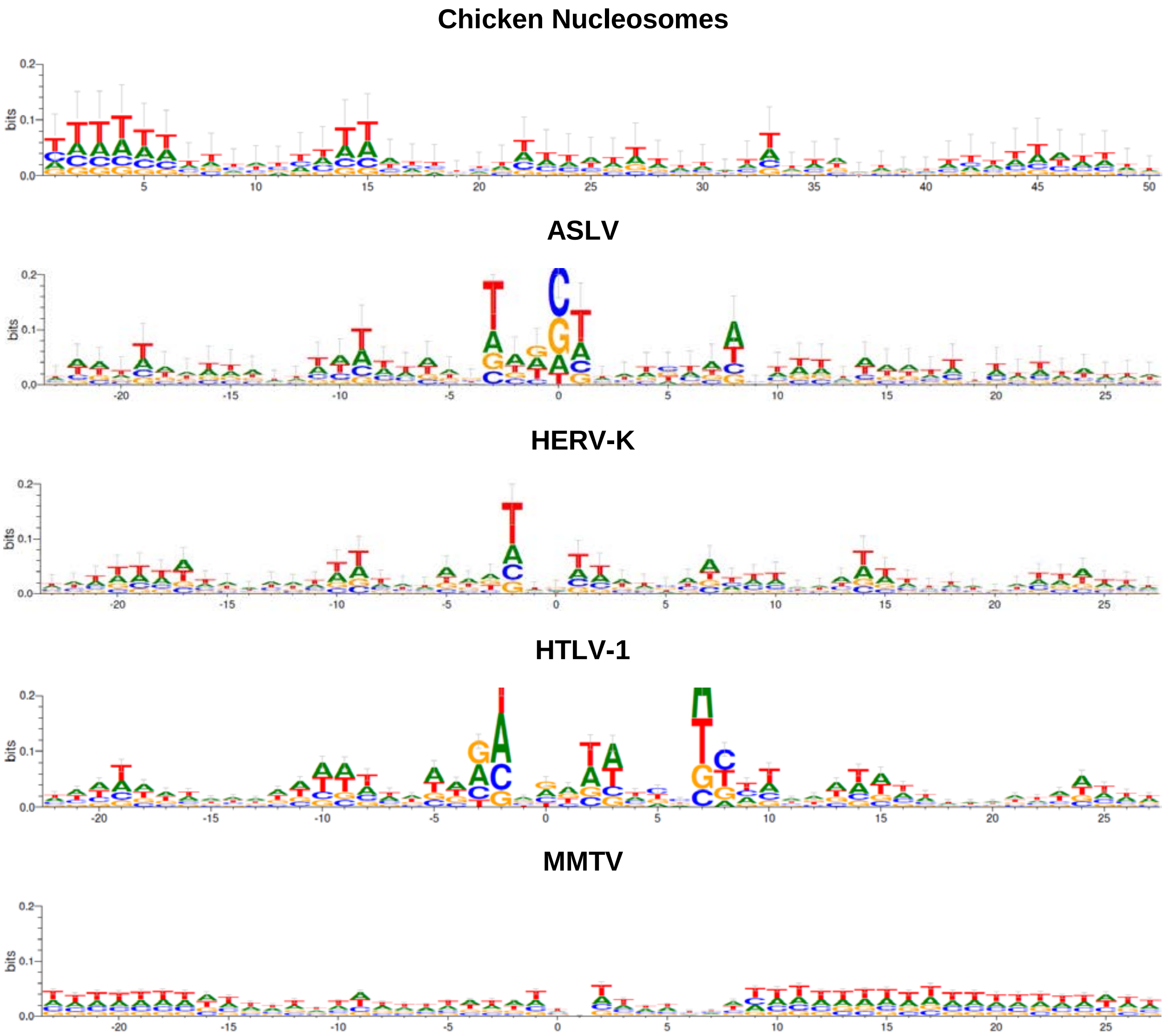

Additional File 7: FIGURE S6 (page 3 of 3)
