# Supplementary material for: Key determinants of target DNA recognition by retroviral intasomes
Source: Retrovirology. 2015 Apr 30;12:39. doi: 10.1186/s12977-015-0167-3 (PMC4422553; doi:10.1186/s12977-015-0167-3)
Supplement: Additional file 8: Figure S7. — Amino acid sequence alignment of CCD α2 helix residues for the viruses analyzed in this study. The location of secondary structural elements has been documented crystallographically for HIV-1 [89], ASLV [90], SIV [91], and PFV [2] INs. Residues analogous to Ala188 in PFV and Ser119 in HIV-1 INs are highlighted in yellow. The active site residue that is analogous to Asp185 in PFV IN and Asp116 in HIV-1 IN is in red type. [file 12977_2015_167_MOESM8_ESM.pdf]

```
                    β4*     α2      α3
PFV      179 PKVIHSDQGAAFTSSTFAEF 198
MoMLV    178 PQVLGTDNGPAFVSKVSQSV 197
PERV     176 PKVIGSDNGPAFVAQVSQGL 195
XMRV     178 PQVLGSDNGPAFASQVSQSV 197
EIAV     110 PKSLHTDNGTNFVAEPVVNL 129
HIV-1    110 VKTVHTDNGSNFTSTTVKAA 129
REV-A    176 PVQIGSDNGPAFVAKVTQQL 195
SIV      110 VKHIHTDNGPNFISKDVEAV 129
ASLV     115 PKAIKTDNGSCFTSKSTREW 134
HERV-K   117 PEKIKTDNGPGYCSKAFQKF 136
HTLV-1   120 PSYINTDNGPAYISQDFLNM 139
MMTV     119 PQKIKTDNAPAYVSRSIQEF 138
```

CCD

Additional File 8: FIGURE S7
